# Supplementary material for: Exploring psychiatrists’ perspectives of working with patients with dissociative seizures in the UK healthcare system as part of the CODES trial: a qualitative study
Source: BMJ Open. 2019 May 9;9(5):e026493. doi: 10.1136/bmjopen-2018-026493 (PMC6528023; doi:10.1136/bmjopen-2018-026493)
Supplement: Supplementary file 1 [file bmjopen-2018-026493supp001.pdf]

Supplementary file

Psychiatrists' interview schedule from which current themes were elicited (material related to the conduct of the CODES Trial to be reported elsewhere)

Psychiatrists Interview Schedule

Section 1: Background and Specific Issues relating to the CODES RCT

How does CODES standardised medical care (SMC) differ from the techniques you would usually use to treat patients with DS?

If SMC is shown to reduce seizures, what do you think will be most difficult about making SMC standard across services?

Were there any parts of the CODES SMC approach that proved more challenging?

How did you manage significant deterioration in a participant's mental health during their time in the CODES study?

How did you feel about NOT referring to other types of therapy whilst a participant was in CODES?

Section 2: Experience of the Intervention

Did the way patients engaged with standardised medical care (SMC) seem to change over time?

Were there any 'lightbulb moments' in the course of SMC where patients appeared to have a sudden understanding of their condition?

Section 3: Individual Psychological, Social or Health-Related Differences and Impact on Treatment

Do you think that there were any factors that may have affected patients understanding of their diagnosis?

Were there any patients who may have been more suitable than others to receive SMC alone? If so, what distinguished these types of clients?

Were there issues that you had to address in order to improve engagement? Or were there any barriers to patients engaging with SMC?

Could sessions ever become side-tracked/derailed by other issues? E.g.: social issues, safeguarding or health-related concerns?
