# Supplementary material for: Exploring psychiatrists’ perspectives of working with patients with dissociative seizures in the UK healthcare system as part of the CODES trial: a qualitative study
Source: BMJ Open. 2019 May 9;9(5):e026493. doi: 10.1136/bmjopen-2018-026493 (PMC6528023; doi:10.1136/bmjopen-2018-026493)
Supplement: Author's manuscript [file bmjopen-2018-026493.draft_revisions.pdf]

BMJ Open is committed to open peer review. As part of this commitment we make the peer review history of every article we publish publicly available.

When an article is published we post the peer reviewers' comments and the authors' responses online. We also post the versions of the paper that were used during peer review. These are the versions that the peer review comments apply to.

The versions of the paper that follow are the versions that were submitted during the peer review process. They are not the versions of record or the final published versions. They should not be cited or distributed as the published version of this manuscript.

BMJ Open is an open access journal and the full, final, typeset and author-corrected version of record of the manuscript is available on our site with no access controls, subscription charges or pay-per-view fees (http://bmjopen.bmj.com).

If you have any questions on BMJ Open's open peer review process please email editorial.bmjopen@bmj.com

BMJ Open

Exploring psychiatrists' perspectives of working with patients with dissociative seizures in the UK healthcare system – A qualitative study

Journal:	*BMJ Open*
Manuscript ID	bmjopen-2018-026493
Article Type:	Research
Date Submitted by the Author:	04-Sep-2018

Complete List of Authors:	Jordan, Harriet; Institute of Psychiatry, Psychology, and Neuroscience, King's College London, De Crespigny Park, London, SE5 8AF, Psychology<br>Feehan, Sarah; Institute of Psychiatry, Psychology, and Neuroscience, King's College London, De Crespigny Park, London, SE5 8AF, Psychology<br>Perdue, Iain; Institute of Psychiatry, Psychology, and Neuroscience, King's College London, De Crespigny Park, London, SE5 8AF, Psychology<br>Murray, Joanna; Institute of Psychiatry, Psychology and Neuroscience, King's College London, Health Service and Population Research<br>Goldstein, Laura; Institute of Psychiatry, Psychology, and Neuroscience, King's College London, De Crespigny Park, London, SE5 8AF, Psychology

Keywords:	Dissociative seizures, psychiatrists, qualitative

SCHOLARONE™
Manuscripts

Exploring psychiatrists' perspectives of working with patients with dissociative seizures in the UK healthcare system – A qualitative study

Harriet Jordan MSc [1], Sarah Feehan BSc [1], Iain Perdue MPhil [1], Joanna Murray BA[2] & Laura H. Goldstein PhD FBPsS [1]

[1] King's College London, Department of Psychology, Institute of Psychiatry, Psychology and Neuroscience, London, UK.

[2] King's College London, Department of Health Service and Population Research, Institute of Psychiatry, Psychology and Neuroscience, London, UK.

Corresponding author: Prof Laura H. Goldstein, Department of Psychology, Institute of Psychiatry, Psychology, and Neuroscience, King's College London, PO77, De Crespigny Park, London, SE5 8AF, UK. Tel ++44 (0)20 7848 0218. laura.goldstein@kcl.ac.uk

Orcid ID:
Harriet Jordan https://orcid.org/0000-0002-7268-7310
Sarah Feehan https://orcid.org/0000-0003-3986-2698
Iain Perdue https://orcid.org/0000-0002-8078-8146
Laura H Goldstein https://orcid.org/0000-0001-9387-3035

Abstract 298 words
Text word count: 3837

Key words: Dissociative seizures; psychiatrists; qualitative.

For peer review only

ABSTRACT

Objective: There is currently limited research exploring healthcare professionals' (HCPs) experiences of working with patients with dissociative seizures (DS). Existing studies do not focus on the role of psychiatrists in treating this complex condition. The objective of this study was to gain an understanding of United Kingdom (UK) based psychiatrists' experiences of the DS patient group. Against the backdrop of a UK-wide randomised controlled trial (RCT), focus was broadened to encompass issues arising in everyday practice with the DS patient group.

Design, Participants and Methods: A qualitative study using semi-structured interviews was undertaken with 10 psychiatrists currently working with DS patients within the context of a large RCT investigating treatments for DS. Thematic analysis was used to identify key themes and subthemes.

Setting: The psychiatrists were working in Liaison or Neuropsychiatry services in England.

Results: The key themes identified were other HCPs' attitudes to DS and the challenges of the DS patient group. There is a clear knowledge gap regarding DS for many HCPs and other clinical services can be reluctant to take referrals for this patient group. Important challenges posed by this patient group included avoidance (of difficult emotions and help), alexithymia and interpersonal difficulties. Difficulties with alexithymia meant DS patients could struggle to identify triggers for their seizures and to express their emotions. Interpersonal difficulties raised included difficulties in attachment with both HCPs and family members.

Conclusions: A knowledge gap for HCPs regarding DS has been identified and needs to be addressed to improve patient care. Given the complexity of the patient group and that clinicians from multiple disciplines will come into contact with DS patients, it is essential for any educational strategy to be implemented across the whole range of specialties, and to account for those already in practise as well as future trainees.

Article summary:

Strengths and limitations of this study

- This study uniquely explores the experiences of psychiatrists providing healthcare to patients with dissociative seizures.

For peer review only - http://bmjopen.bmj.com/site/about/guidelines.xhtml

- The findings have implications for guidance on interventions for people with dissociative seizures, specifically in relation to epilepsy.

- The study has a small sample size of 10 psychiatrists. The psychiatrists were all currently working at healthcare centres across England.

- Psychiatrists working with DS patients in Scotland and Wales are not part of our sample.

For peer review only

For peer review only - http://bmjopen.bmj.com/site/about/guidelines.xhtml

BACKGROUND

Dissociative seizures (DS) (often also referred to as Psychogenic Nonepileptic Seizures (PNES), Non-Epileptic Attack Disorder (NEAD), or functional seizures) are similar in appearance to epileptic seizures without the abnormal neural activity. The incidence of DS is reported as approximately 4.9 per 100,000/per year, [1] with some estimates reaching as high as 50 per 100,000/per year.[2] DS are a common challenge in epilepsy centres worldwide, [3 4] with between 12 and 20% of patients referred for telemetry having coexisting or misdiagnosed DS. [5 6]

Quantitative research has indicated that there is a gap in the knowledge of healthcare professionals (HCPs) regarding DS[7] and that some HCPs have a negative attitude towards patients with DS, perceiving the seizures as being under their control and seeing DS as untreatable. [8 9 10] Patients often describe feeling hopeless [11] and negative experiences with HCPs are frequently reported. [12] Previous research has found that clinicians, including general practitioners (GPs), have felt uncertainty in treating patients with DS [13 14] due to the lack of substantial evidence-base for any one particular intervention.

The CODES (COgnitive behavioural therapy vs standardised medical care for adults with Dissociative non-Epileptic Seizures) Trial is the first sufficiently powered multi-centre, pragmatic, parallel group randomised controlled trial (RCT) to investigate the effectiveness of any psychological therapy for patients with DS. CODES is evaluating the clinical effectiveness and cost-effectiveness of cognitive behavioural therapy (CBT) plus trial standardised medical care (SMC) compared with SMC alone. [15] Each patient recruited into the study was first seen by a neurologist and then referred on to a psychiatrist for assessment, despite this care pathway not being widely followed outside of the CODES Trial in some areas of the United Kingdom (UK) within the context of the National Health Service (NHS).

When evaluating complex interventions such as those tested in the CODES Trial, it can be difficult to capture effectiveness using only quantitative methods.[16] Research has found that combining quantitative and qualitative methods provides essential insight into how and why an intervention is effective, if at all. [17 18] The purpose of this study was to gain an understanding of attitudes and beliefs among psychiatrists who had been part of the CODES Trial and were experienced in working with patients with DS, with particular emphasis on psychiatrists' ability and willingness to work with DS patients in the context of the NHS.

For peer review only

For peer review only - http://bmjopen.bmj.com/site/about/guidelines.xhtml

METHODS

Study population

Ten participants were purposively selected from the 27 psychiatrists involved in the CODES RCT to encompass the geographical distribution of the CODES Trial and the range of experience treating functional neurological disorders (FNDs), particularly DS. All participants were known to the wider CODES team prior to taking part in this qualitative study. CODES Trial grant holders were excluded to avoid study design-related bias. The psychiatrists were based at nine different NHS Trusts across England and practised in either Liaison or Neuropsychiatry specialties. Recruitment took place between June and September 2017.

Data Collection

Those selected were initially contacted via email by HJ and invited to take part. They were provided with an information sheet and a description of the aims of the project. If they were interested, a work place based face-to-face interview was scheduled at a time and date convenient for them. All participants provided informed consent to be interviewed. All interviews were conducted by HJ and recorded using an encrypted digital voice recorder to ensure data security and confidentiality. Interviews lasted between 41 – 96 minutes.

Interview Schedule

The interview schedule was developed by members of the CODES study team of which all authors were a part. The topics covered experiences of delivering the CODES SMC and involvement in the CODES RCT more generally (which will be reported elsewhere). In addition, topics covered the delivery of diagnosis, DS in the context of the NHS, and the challenges of the patient group, which are the focus of this paper. Participants were encouraged to give examples where possible and probing techniques were used to explore responses and elicit further detail where necessary [19] (see Appendix A). The interview began with a series of questions about aspects of the trial processes which will be reported elsewhere.

Data Analysis

The interviews were transcribed verbatim by members of the CODES research team. During the transcription process the interview data was anonymised. Completed transcripts were checked by HJ against the original recordings to ensure accuracy. The semi-structured interviews were analysed using thematic analysis. [20] Three of the completed transcripts were chosen at random and coded

For peer review only - http://bmjopen.bmj.com/site/about/guidelines.xhtml

initially by HJ, SF and another member of the research team. Emerging findings and preliminary themes were discussed in team meetings. HJ and SF then coded all 10 transcripts independently, using the qualitative data analysis software NVivo 11 (QSR International). Coding was done independently to allow for an organic and reflexive process. Themes were identified that were representative of the content. As each interview was analysed, new themes were added to the list. Regular meetings were held to discuss agreements in coding and establish the parameters of each major theme. Major themes that had been identified by both coders were then combined, with subthemes being organized under the appropriate main theme.

Patient and public involvement

The CODES Trial has service user involvement in its management committees. The paper also underwent service user review.

RESULTS

Interviews from the 10 psychiatrists were analysed (see Table 1 for the psychiatrists' demographic characteristics). In general, there was a high level of agreement among participants on most topics covered. This made it straightforward to identify main themes and clearly convey the conclusions drawn from the clinicians. Though the topic guide elicited a broad range of themes, for the purposes of this paper we focused on those that had significant clinical implications. Other themes relating to the CODES Trial will be described elsewhere. Two main clinically relevant overarching themes emerged from the data: i) Other Healthcare Professionals and DS and (ii) Psychiatrists' Identified Challenges of Working with DS.

For peer review only - http://bmjopen.bmj.com/site/about/guidelines.xhtml

Table 1 *Psychiatrists' self-reported demographic information*					
	**N**	**%**		**N**	**%**
**Age**			**Sub Specialist Accreditation**		
31-40	1	10%	Liaison Psychiatry	6	60%
41-50	8	80%	Neuropsychiatry	1	10%
51-60	1	10%	Both	3	30%
**Gender**			**Years of Experience**		
Female	5	50%	11-15	5	50%
Male	5	50%	16-20	2	20%
			21-25	1	10%
**Location**			26-30	2	20%
London	6	60%			
Rest of England	4	40%			

Other Healthcare Professionals (HCPs) and DS

HCPs ill-equipped to deal with DS

Psychiatrists reported that HCPs from other services often felt uncertain when dealing with DS patients or were not prepared to work with patients with functional neurological symptoms. Others felt that DS is a disorder that GPs should be able to work with. It was also reported that services would often contribute to the diagnostic confusion by continually mistaking DS for epilepsy, despite referrals stating otherwise;

> *"They would come back saying well, look, this is epilepsy, they need to be seeing a neurologist, or people would end up back on anticonvulsants"*
> *(Psychiatrist 09, Female, Liaison Psychiatry).*

The mention of seizures would often result in a panicked response from some primary care psychology services that meant patients could sense having something difficult to treat. Psychiatrists described patients often feeling other clinicians had not given a positive message about a DS diagnosis, with some GPs reportedly stating the need to be on an anticonvulsant simply at the mention of seizures. This continual reference to epilepsy by other professionals can have a negative impact on patients' progress,

For peer review only - http://bmjopen.bmj.com/site/about/guidelines.xhtml

"They have said, oh this sounds…you have epilepsy. I say don't say that, you're not qualified to say that, you know, you do your job, uh because that one word would put patients (pause) back, by a year or two or ten sessions."

(Psychiatrist 06, Male, Neuropsychiatry)

Psychiatrists would find that making DS referrals to psychology services would result in the referral being rejected unless patients had a comorbidity that psychologists felt they could treat;

"So, if you send a referral saying this person has dissociative seizures, will you see them, they will return the referral, so you have to say, "this person has dissociative seizures; however, they also have a very clear anxiety or panic disorder and that is what I would like you to work on" and then they will accept it."

(Psychiatrist 03, Female, Liaison Psychiatry)

This was reiterated throughout most of the interviews, with psychiatrists stating that local services would prefer to treat comorbidities rather than the DS themselves and where no comorbidity could be identified, services often rejected the referrals. The majority of the interviewees endorsed the view that psychiatrists were a key part of DS patient care. However, two of the 10 questioned whether it was necessary in all cases for a psychiatrist to be involved especially if the DS patient had no clear psychiatric co-morbidities. This approach seemed to be influenced both by their usual practice and service pressures at the two trusts.

The need for experience

One conclusion frequently drawn from psychiatrists' experiences with HCPs in other services was that, in order to diagnose and treat DS, the clinician needed to have a significant level of experience with the disorder and that treatment should be undertaken in a specialist setting,

"I sincerely believe that…it's not a condition which anybody or everybody can deal with and I don't think it should be dealt with at IAPT level"

(Psychiatrist 06, Male, Neuropsychiatry). (IAPT = Improving Access to Psychological Therapies services; the IAPT programme began in 2008 and aims to offer short-term evidence based psychological treatments for depression and anxiety in adults across England).

For peer review only - http://bmjopen.bmj.com/site/about/guidelines.xhtml

Delivering treatment in a specialist centre was described as not only helpful in terms of clinicians knowing how to work with DS, but would provide reassurance for patients that they were being seen by someone who is confident and experienced;

> *"I think it's one of those conditions where seeing people who know what it is, know what to do with it even if they can't promise to get it better it reduces everybody's anxiety levels about it"*
> *(Psychiatrist 09, Female, Liaison Psychiatry).*

This sense of needing experience was also reported as helpful in enabling professionals to acknowledge the amount that can often be unknown about the causes and triggers for DS and for helping the patient embrace that as well.

Psychiatrists' Identified Challenges of Working with DS

Avoidance

Avoidance was viewed as a key area of difficulty for the DS patient group and was noted to take a number of forms across 9/10 interviews. A number of examples of avoidance were given in the interviews but these seem to fall into two broad categories: "avoidance of help" and "avoidance of emotions". Avoidance of help included not reading information about DS even when handed this directly in an appointment and avoiding attending medical or therapy appointments. Avoidance of emotions included a desire to take medication rather than deal with difficult feelings and blocking emotions;

> *"Quite a lot of people may have blocked out so to speak, the more emotional side of things and try to get on with things…. if they start to go through a more open exploration of the issues this can be very emotionally distressing and suddenly their mood goes down…… a lot of patients will have to go through that turbulence in order to come out on the other end having felt the issues, recognised them and dealt with it"*
> (Psychiatrist 06, Male, Neuropsychiatry)

The seizures themselves were seen as potentially fitting into a pattern of avoidant behaviour as a defence against difficult emotions. Avoidant behaviour could present under the guise of other difficulties such as a reluctance to travel to appointments.

For peer review only - http://bmjopen.bmj.com/site/about/guidelines.xhtml

Linked to the theme of avoidance was emotional literacy. This was commented on by 9/10 interviewees as a key difficulty for the DS patient group. It follows that if a person lacks awareness of their own emotions they would struggle to express these to others. This lack of emotional awareness could then impede the ability to make links between life events and feelings whilst in treatment.

> *"No symptoms, happy go lucky kind of personality, I love my family, no trauma, no pain and those people are the hardest*"
> (Psychiatrist 10, Female, Liaison Psychiatry)

Some of the most challenging of the DS patient group were those for whom no trigger for the seizures could be identified. Sometimes even when it was very clear to the clinician that there was a current stressor (such as caring for a gravely ill partner) patients with DS might deny this was the case. This seemed to lead to feelings of frustration for the psychiatrists as they viewed patients with no identified psychological trigger harder to treat successfully.

Complex interpersonal relationships

Eight out of the 10 psychiatrists noted that a patient with DS may well struggle with relationships, both with people generally and within the clinician-patient relationship. This could be associated with difficulties in attachment with the DS patient becoming over-attached and then not wanting to engage with any other clinician or be discharged.

> *"She became quite attached; there were real attachment issues…. so, I only managed to discharge her as I said a few weeks ago…… she didn't connect with that person [CODES CBT therapist] and she created a split between that person and me and she was like I just want to come and see you, can't I just come and see you every two weeks*?"
> (Psychiatrist 08, Female Liaison Psychiatry)

Attempts at splitting were described as occurring not only between individual clinicians (as above) but also between services e.g., a specialist national service being "good" and all local services being "bad". This splitting and idealisation of one service or clinician could be accompanied by unrealistic expectations that the psychiatrist would continue to see them indefinitely. A partner being in the room throughout every appointment can mean the patient is less able to be open about how they are feeling and certainly less likely to discuss any current relationship difficulties. A co-dependent relationship may also impact on motivation to recover as some people with DS have social

For peer review only - http://bmjopen.bmj.com/site/about/guidelines.xhtml

circumstances in which getting better may feel riskier than remaining unwell. A couple of the psychiatrists went further and conceptualised some examples of factitious behaviours in patients (e.g. deliberately concealing medication non-adherence from family and clinicians) as driven by a profound need to be looked after.

DISCUSSION

We present here the experiences and views of the psychiatrists involved in an RCT for patients with DS. The characteristics of the nested group were similar to the clinicians involved in the trial as a whole in relation to age, gender, ethnicity and experience working with DS. Participants generally expressed concordant views across the range of interview questions, suggesting that issues surrounding DS are very apparent to the professionals working closely with this population. Views broadly support previous research that describes DS patients as a heterogeneous population with complex presentations and demanding HCP input,[8 10 21] as well as the need for an improvement in education, awareness of DS by HCPs.[22]

Participants believed that interventions by other HCPs at times made their own work with this patient group more difficult. They identified a knowledge gap surrounding DS among other HCPs. Previous research has found that HCPs from a variety of backgrounds often have very different perceptions regarding DS.[23] This seems to be a pervasive problem at all levels of health services in the UK, from GPs and primary care services to Community Mental Health Trusts and Emergency Departments. What is perhaps most significant is the participants' observation that this knowledge gap can at times have a detrimental effect on patients' prognosis, with one participant noting that the mention of epilepsy and AED treatment can set a patient's progress back significantly. Rawlings and Reuber's recent review[10] raised concerns not only about HCPs' DS knowledge gap but also negative attitudes towards the condition. It is of concern that negative attitudes towards the DS patient group may be created or reinforced by HCPs currently in practice when training junior staff.[10] Dworetzky[30] reported that epilepsy specialists in the USA when teaching junior staff about DS tend to focus on the cost of care and misuse of services caused by DS. Negative clinician attitudes did not emerge as a theme here, perhaps because our group of psychiatrists have chosen to work with this patient group.

For peer review only - http://bmjopen.bmj.com/site/about/guidelines.xhtml

Referring to epilepsy can be damaging to patients with DS as it contributes to diagnostic confusion in a number of ways. If they have been diagnosed with DS already, it may cause them to doubt whether the diagnosis is correct. It may initiate or strengthen a belief that they in fact have epilepsy, despite diagnostic evidence (e.g.; video-electroencephalography (vEEG) telemetry results) and clinical opinion to the contrary. [24] It also means that often DS patients are treated with potentially harmful anti-epileptic drugs (AEDs) with serious side effects despite them having no medical benefit and this can lead to serious iatrogenic harm. [25] It would be helpful for more specific epilepsy-related guidance to be developed for HCPs with regards to reducing AEDs and handling a misdiagnosis of epilepsy so as to avoid any setbacks in recovery. HCPs should be made aware of the clinical significance that simply mentioning epilepsy and AEDs can have on a patient and this should be highlighted in any future educational resources.

The International League Against Epilepsy (ILAE) PNES Task Force produced a special report describing the minimum requirements for a diagnosis of DS[26]. It would be beneficial for those working with DS to become familiar with these guidelines so that they can clearly convey their diagnostic reasoning to the patient and can discuss the features of DS and the characteristics distinguishing DS from epilepsy more confidently. In addition to this detailed account of the diagnosis, LaFrance et al. [24] also produced a comprehensive overview of the management of DS patients, encompassing diagnosis, treatment and maintaining engagement. Whilst it is argued that the management of DS requires expertise, the guidelines are accessible and can be used as a helpful tool for non-experts to familiarize themselves with the important elements for interacting with this patient group.

There were some discordant views surrounding which services and professionals should be able to work with DS. Some participants described the need for more specialist services to be made available and for psychiatric training to cover functional neurological symptoms in greater depth. However, some participants stressed the necessity for GPs and primary care clinicians to also become more familiar with DS. Despite this contrast, the consensus remains that more education and awareness are required for all HCPs regardless of their speciality. This supports previous research that has reported the need for more clinicians to be comfortable with treating all FNDs, particularly DS. [27] Encouragingly going forward even a brief training intervention for medical students and doctors' improved confidence levels and diagnostic accuracy when working with DS. [28 29]

For peer review only - http://bmjopen.bmj.com/site/about/guidelines.xhtml

To address this issue, better resources and educational materials need to be made available for those who are likely to be working with DS, both for future clinicians and clinicians currently in practice. Rommelfanger et al. [22] described a level of "professional isolation" often felt among care providers working with functional symptoms due to a distinct lack of formalised training and reported on the need for a shift in priorities to support clinicians working with patients with FNDs. This could start as early as medical school but should also involve the development of sufficient resources to support the multidisciplinary approach that is often required to treat FNDs.

Avoidant behaviours could be divided into two categories of "avoidance of help" and "avoidance of emotions". Linked to avoidance was the subtheme of emotional literacy, difficulty feeling and expressing emotions. These findings support previous quantitative research on alexithymia in patients with DS, where Bewley et al. [31] found DS patients had significantly greater difficulty identifying feelings than healthy controls. More recently, Uliaszek et al. [32] found significant emotion dysregulation among DS patients when compared with a control group. This may have important implications for future therapeutic developments, by incorporating elements of effective treatments for alexithymia from other treatment models (e.g. mindfulness-based therapy for alexithymia). [33] Previous research also found evidence of experiential avoidance [34] and avoidant coping styles [35] among the DS patient population. Given these findings, it is important to establish what impact this avoidant behaviour may have on treatment outcomes and what can be done to mitigate any negative impacts. Some participants in the present study reported positive progress in openly identifying the patients' avoidant behaviour and providing the opportunity to address it constructively. Treatment approaches for DS should make provision for tackling avoidance directly. [15 36] In addition, for a subset of the patient group, no trigger for DS is identified and these patients tended to be particularly difficult to treat from the point of explaining the diagnosis onwards. Working with this complex patient group effectively clearly needs experience and knowledge of the condition.

Our study has a number of limitations. The study has a small sample size of 10 psychiatrists currently working at healthcare centres around England. Psychiatrists working in Scotland and Wales are not represented in this group. It is possible that the DS patient sample in the CODES Trial, who will have partly influenced the current psychiatrists' views, is not fully representative of the DS patient population in general. It is also likely that there is a self-selection bias for those presenting to psychiatrists as many DS patients may reject a psychiatric diagnosis and thus not attend psychiatric appointments. [37] The disparity in services may mean that some patients simply do not have access to

For peer review only - http://bmjopen.bmj.com/site/about/guidelines.xhtml

a specialist psychiatrist and are referred back to their GP. [38] We will report elsewhere an exploration of the views of patients with DS involved in the CODES Trial to triangulate findings and maximise our understanding of working and living with DS.

In terms of strengths, to our knowledge, our study was the first qualitative study focusing solely on psychiatrists' perspectives of working with patients with DS. Prior published qualitative studies [39 40] interviewed healthcare professionals from a variety of backgrounds. However, McMillan et al.'s [39] large sample (74 interviews) only included epilepsy staff such as neurologists, EEG technicians and epilepsy nurses and no mental health care providers. In du Toit & Pretorius's [40] study, only three of 15 people interviewed were psychiatrists. Given that DS is classified as a mental health disorder (DSM5, [41]) and a dissociative disorder (ICD-10 [42]) and will predominantly be handled by mental health clinicians, it seems important to explore the views of those working with the DS population in the appropriate clinical context. In addition, it is likely that the current sample is more representative of the clinicians most likely to be working with DS in developed countries, and not limited to a military clinical environment. [39]

CONCLUSION

Qualitative findings suggest that patients with DS are a complex and at times challenging population that requires intervention from experienced clinicians familiar with the condition. Significantly, intervention can be made more difficult if not provided in an informed and experienced manner. Our findings have important implications for medical and allied professional training with regards to FNDs in order for clinicians to be better equipped to recognise and handle the challenges that come with treating DS. It is also hoped that a greater evidence base for treatments for patients with DS will help eradicate the variability within healthcare provision.

Acknowledgements: The authors would like to thank the psychiatrists who were willing to be interviewed for this study given their busy schedules. The authors also thank Julie Read for her assistance with the project.

Author contributions: HJ, IP, JM and LHG contributed to the development of the topic guide. HJ carried out all data collection. HJ and SF transcribed the data, carried out the analysis and led the

For peer review only - http://bmjopen.bmj.com/site/about/guidelines.xhtml

writing of the paper. JM and IP reviewed drafts of the paper. LHG oversaw the project, contributed to the writing of the paper and reviewed successive drafts. All authors approved the final version of the submitted text.

Funding statement: This paper describes independent research funded by the National Institute for Health Research (Health Technology Assessment programme, 12/26/01, COgnitive behavioural therapy vs standardised medical care for adults with Dissociative non-Epileptic Seizures: A multicentre randomised controlled trial (CODES)). LHG receives salary support from the National Institute for Health Research (NIHR) Maudsley Biomedical Research Centre at the South London and Maudsley NHS Foundation Trust and King's College London. The views expressed in this publication are those of the authors and not necessarily those of the NHS, the NIHR or the Department of Health.

Competing interests: LHG and JM report the grant from the NIHR HTA for the conduct of the study. None of the other authors have competing interests to declare.

Data sharing statement: Contact the corresponding author with queries.

Ethics approval: The study was approved by the NHS Health Research Authority, London – Camberwell St Giles Research Ethics Committee (reference number 13/LO/1595). Written informed consent was obtained from all study participants. Study registration: Current Controlled Trials: ISRCTN05681227; ClinicalTrials.gov: NCT02325544.

For peer review only

APPENDIX

Psychiatrists' interview schedule from which current themes were elicited (material related to the conduct of the CODES Trial to be reported elsewhere)

Psychiatrists Interview Schedule
**Section 1: Background and Specific Issues relating to the CODES RCT**<br><br>How does CODES standardised medical care (SMC) differ from the techniques you would usually use to treat patients with DS?<br><br>If SMC is shown to reduce seizures, what do you think will be most difficult about making SMC standard across services?<br><br>Were there any parts of the CODES SMC approach that proved more challenging?<br><br>How did you manage significant deterioration in a participant's mental health during their time in the CODES study?<br><br>How did you feel about NOT referring to other types of therapy whilst a participant was in CODES?<br><br>**Section 2: Experience of the Intervention**<br><br>Did the way patients engaged with standardised medical care (SMC) seem to change over time?<br><br>Were there any 'lightbulb moments' in the course of SMC where patients appeared to have a sudden understanding of their condition?<br><br>**Section 3: Individual Psychological, Social or Health-Related Differences and Impact on Treatment**<br><br>Do you think that there were any factors that may have affected patients understanding of their diagnosis?<br><br>Were there any patients who may have been more suitable than others to receive SMC alone? If so, what distinguished these types of clients?<br><br>Were there issues that you had to address in order to improve engagement? Or were there any barriers to patients engaging with SMC?<br><br>Could sessions ever become side-tracked/derailed by other issues? E.g.: social issues, safeguarding or health-related concerns?

For peer review only - http://bmjopen.bmj.com/site/about/guidelines.xhtml

REFERENCES

1. Duncan R, Razvi S, Mulhern S. Newly presenting psychogenic nonepileptic seizures: Incidence, population characteristics, and early outcome from a prospective audit of a first seizure clinic. *Epilepsy Behav* 2011;20(2):308-11. doi: 10.1016/j.yebeh.2010.10.022
2. Kanemoto K, LaFrance WC, Duncan R, et al. PNES around the world: Where we are now and how we can close the diagnosis and treatment gaps—an ILAE PNES Task Force report. *Epilepsia Open* 2017;2(3):307-16. doi: doi:10.1002/epi4.12060
3. Asadi-Pooya AA, Sperling MR. Epidemiology of psychogenic nonepileptic seizures. *Epilepsy Behav* 2015;46:60-65. doi: https://doi.org/10.1016/j.yebeh.2015.03.015
4. Kerr MP, Mensah S, Besag F, et al. International consensus clinical practice statements for the treatment of neuropsychiatric conditions associated with epilepsy. *Epilepsia* 2011;52(11):2133-38. doi: 10.1111/j.1528-1167.2011.03276.x
5. Angus-Leppan H. Diagnosing epilepsy in neurology clinics: A prospective study. *Seizure* 2008;17(5):431-36. doi: 10.1016/j.seizure.2007.12.010
6. Lesser RP. Psychogenic seizures. *Neurology* 1996;46(6):1499-507.
7. Worsely C, Whitehead K, Kandler R, et al. Illness perceptions of health care workers in relation to epileptic and psychogenic nonepileptic seizures. *Epilepsy Behav* 2011;20(4):668-73. doi: https://doi.org/10.1016/j.yebeh.2011.01.029
8. Hingray C, El-Hage W, Duncan R, et al. Access to diagnostic and therapeutic facilities for psychogenic nonepileptic seizures: An international survey by the ILAE PNES Task Force. *Epilepsia* 2018;59(1):203-14. doi: doi:10.1111/epi.13952
9. Kanaan RA, Armstrong D, Wessely SC. Neurologists' understanding and management of conversion disorder. *J Neurol Neurosurg Psychiatry* 2011;82(9):961-6. doi: http://dx.doi.org/10.1136/jnnp.2010.233114
10. Rawlings GH, Reuber M. Health care practitioners' perceptions of psychogenic nonepileptic seizures: A systematic review of qualitative and quantitative studies. *Epilepsia* 2018;59(6):1109-23. doi: doi:10.1111/epi.14189
11. Karterud HN, Knizek BL, Nakken KO. Changing the diagnosis from epilepsy to PNES: Patients' experiences and understanding of their new diagnosis. *Seizure* 2010;19(1):40-46. doi: http://dx.doi.org/10.1016/j.seizure.2009.11.001
12. Rawlings GH, Reuber M. What patients say about living with psychogenic nonepileptic seizures: A systematic synthesis of qualitative studies. *Seizure* 2016;41:100-11. doi: http://dx.doi.org/10.1016/j.seizure.2016.07.014
13. Shneker BF, Elliott JO. Primary care and emergency physician attitudes and beliefs related to patients with psychogenic nonepileptic spells. *Epilepsy Behav* 2008;13(1):243-47. doi: https://doi.org/10.1016/j.yebeh.2008.03.001
14. O'Sullivan SS, Sweeney BJ, McNamara B. The opinion of the general practitioner toward clinical management of patients with psychogenic nonepileptic seizures. *Epilepsy Behav* 2006;8(1):256-60. doi: https://doi.org/10.1016/j.yebeh.2005.09.013
15. Goldstein LH, Mellers JDC, Landau S, et al. COgnitive behavioural therapy vs standardised medical care for adults with Dissociative non-Epileptic Seizures (CODES): A multicentre randomised controlled trial protocol. *BMC Neurol* 2015;15(1) doi: http://dx.doi.org/10.1186/s12883-015-0350-0
16. Lewin S, Glenton C, Oxman AD. Use of qualitative methods alongside randomised controlled trials of complex healthcare interventions: methodological study. *BMJ* 2009;339 doi: 10.1136/bmj.b3496
17. Möhler R, Köpke S, Meyer G. Criteria for Reporting the Development and Evaluation of Complex Interventions in healthcare: revised guideline (CReDECI 2). *Trials* 2015;16(1):204. doi: 10.1186/s13063-015-0709-y
18. Campbell M, Fitzpatrick R, Haines A, et al. Framework for design and evaluation of complex interventions to improve health. *BMJ* 2000;321 doi: 10.1136/bmj.321.7262.694

For peer review only - http://bmjopen.bmj.com/site/about/guidelines.xhtml

19. Rubin H, Rubin I. Qualitative interviewing: A method to the madness: Thousand Oaks, CA: Sage Publications, 1995.
20. Braun V, Clarke V. Using thematic analysis in psychology. *Qualitative Research in Psychology* 2006;3(2):77-101. doi: 10.1191/1478088706qp063oa
21. Brown RJ, Syed TU, Benbadis S, et al. Psychogenic nonepileptic seizures. *Epilepsy Behav* 2011;22(1):85-93. doi: 10.1016/j.yebeh.2011.02.016
22. Rommelfanger KS, Factor SA, LaRoche S, et al. Disentangling Stigma from Functional Neurological Disorders: Conference Report and Roadmap for the Future. *Front Neurol* 2017;8(106) doi: 10.3389/fneur.2017.00106
23. Whitehead K, Reuber M. Illness perceptions of neurologists and psychiatrists in relation to epilepsy and nonepileptic attack disorder. *Seizure* 2012;21(2):104-9.
24. Lafrance Jr WC, Reuber M, Goldstein LH. Management of psychogenic nonepileptic seizures. *Epilepsia* 2013;54:53-67. doi: http://dx.doi.org/10.1111/epi.12106
25. Reuber M, Elger CE. Psychogenic nonepileptic seizures: Review and update. *Epilepsy and Behavior* 2003;4(3):205-16.
26. Lafrance Jr WC, Baker GA, Duncan R, et al. Minimum requirements for the diagnosis of psychogenic nonepileptic seizures: A staged approach: A report from the International League Against Epilepsy Nonepileptic Seizures Task Force. *Epilepsia* 2013;54(11):2005-18. doi: http://dx.doi.org/10.1111/epi.12356
27. Gardiner P, MacGregor L, Carson A, et al. Occupational therapy for functional neurological disorders: a scoping review and agenda for research. *CNS Spectrums* 2017;23(3):205-12. doi: 10.1017/S1092852917000797 [published Online First: 11/27]
28. O'Sullivan SS, Redwood RI, Hunt D, et al. Recognition of psychogenic non-epileptic seizures: a curable neurophobia? *Journal of Neurology, Neurosurgery & Psychiatry* 2013;84(2):228-31. doi: 10.1136/jnnp-2012-303062
29. Seneviratne U, Ding C, Bower S, et al. Video-based training improves the accuracy of seizure diagnosis. *Journal of Neurology, Neurosurgery & Psychiatry* 2014 doi: 10.1136/jnnp-2013-306618
30. Dworetzky BA. What Are We Communicating When We Present the Diagnosis of PNES? *Epilepsy Curr* 2015;15(6):353-57. doi: 10.5698/1535-7511-15.6.353
31. Bewley J, Murphy PN, Mallows J, et al. Does alexithymia differentiate between patients with nonepileptic seizures, patients with epilepsy, and nonpatient controls? *Epilepsy and Behavior* 2005;7(3):430-37.
32. Uliaszek AA, Prensky E, Baslet G. Emotion regulation profiles in psychogenic non-epileptic seizures. *Epilepsy Behav* 2012;23(3):364-69. doi: https://doi.org/10.1016/j.yebeh.2012.01.009
33. Norman H, Marzano L, Coulson M, et al. Effects of mindfulness-based interventions on alexithymia: a systematic review. *Evidence Based Mental Health* 2018
34. Dimaro LV, Dawson DL, Roberts NA, et al. Anxiety and avoidance in psychogenic nonepileptic seizures: The role of implicit and explicit anxiety. *Epilepsy Behav* 2014;33:77-86. doi: 10.1016/j.yebeh.2014.02.016
35. Goldstein LH, Drew C, Mellers J, et al. Dissociation, hypnotizability, coping styles and health locus of control: Characteristics of pseudoseizure patients. *Seizure* 2000;9(5):314-22.
36. Goldstein LH, Chalder T, Chigwedere C, et al. Cognitive-behavioral therapy for psychogenic nonepileptic seizures A pilot RCT. *Neurology* 2010;74(24):1986-94.
37. Carton S, Thompson PJ, Duncan JS. Non-epileptic seizures: patients' understanding and reaction to the diagnosis and impact on outcome. *Seizure* 2003;12(5):287-94.
38. Agrawal N, Fleminger S, Ring H, et al. Neuropsychiatry in the UK: National survey of existing service provision. *Psychiatric Bulletin* 2008;32(8):288-91. doi: http://dx.doi.org/10.1192/pb.bp.107.018424

For peer review only - http://bmjopen.bmj.com/site/about/guidelines.xhtml

39. McMillan KK, Pugh MJ, Hamid H, et al. Providers' perspectives on treating psychogenic nonepileptic seizures: Frustration and hope. *Epilepsy and Behavior* 2014;37:276-81. doi: http://dx.doi.org/10.1016/j.yebeh.2014.07.001
40. du Toit A, Pretorius C. Psychogenic nonepileptic seizures: Namibian healthcare providers' perceptions and frustrations. *Seizure* 2017;50:43-52. doi: https://doi.org/10.1016/j.seizure.2017.06.004
41. American Psychiatric Association. Diagnostic and statistical manual of mental disorders Fifth edition. United States: American Psychiatric Association; 2013.
42. World Health Organization. The ICD-10 Classification of Mental and Behavioural Disorders: Clinical Descriptions and Diagnostic Guidelines: World Health Organization 1992.

For peer review only

For peer review only - http://bmjopen.bmj.com/site/about/guidelines.xhtml

Consolidated criteria for reporting qualitative studies (COREQ): 32-item checklist

Developed from:
Tong A, Sainsbury P, Craig J. Consolidated criteria for reporting qualitative research (COREQ): a 32-item checklist for interviews and focus groups. *International Journal for Quality in Health Care*. 2007. Volume 19, Number 6: pp. 349 – 357

YOU MUST PROVIDE A RESPONSE FOR ALL ITEMS. ENTER N/A IF NOT APPLICABLE

No. Item	Guide questions/description	Reported on Page #
**Domain 1: Research team and reflexivity**		
Personal Characteristics		
1. Inter viewer/facilitator	Which author/s conducted the **interviews** or focus group?	Methods, Page 5
2. Credentials	What were the researcher's credentials? E.g. PhD, MD	Authors, Page 1
3. Occupation	What was their occupation at the time of the study?	Methods, Page 5, where we state that The interview schedule was developed by members of the CODES study team, of which all authors were members.
4. Gender	Was the researcher male or female?	Authors list. Page 1
5. Experience and training	What experience or training did the researcher have?	Author JM is a qualitative research expert and supervised the project.
Relationship with participants		
6. Relationship established	Was a relationship established prior to study commencement?	Member of the CODES study research team had prior involvement with participants, as all interviewees are involved in this study. Participants, Page 5.
7. Participant knowledge of the interviewer	What did the participants know about the researcher? e.g. personal goals, reasons for doing the research	See above.
8. Interviewer characteristics	What characteristics were reported about the inter viewer/facilitator? e.g. Bias, assumptions, reasons and interests in the	N/A We have not reported this as we are not undertaking

For peer review only - http://bmjopen.bmj.com/site/about/guidelines.xhtml

	research topic	IPA.
**Domain 2: study design**		
Theoretical framework		
9. Methodological orientation and Theory	What methodological orientation was stated to underpin the study? e.g. grounded theory, discourse analysis, ethnography, phenomenology, content analysis	Thematic Analysis. Methods, Page 5.
Participant selection		
10. Sampling	How were participants selected? e.g. **purposive**, convenience, consecutive, snowball	Methods, Page 5.
11. Method of approach	How were participants approached? e.g. face-to-face, telephone, mail, **email**	Methods, Page 5
12. Sample size	How many participants were in the study?	Methods, Page 5
13. Non-participation	How many people refused to participate or dropped out? Reasons?	No refusals. N/A.
Setting		
14. Setting of data collection	Where was the data collected? e.g. home, clinic, workplace	Face to face. Methods, page 5
15. Presence of non-participants	Was anyone else present besides the participants and researchers?	N/A
16. Description of sample	What are the important characteristics of the sample? e.g. demographic data, date	Results, Demographic Table Page 6
Data collection		
17. Interview guide	Were questions, prompts, guides provided by the authors? Was it pilot tested?	Methods, page 5 Prompts & Probes used. Not pilot tested.
18. Repeat interviews	Were repeat inter views carried out? If yes, how many?	No repeat interviews conducted. N/A.
19. Audio/visual recording	Did the research use audio or visual recording to collect the data?	Methods, Page 5
20. Field notes	Were field notes made during and/or after the inter view or focus group?	N/A
21. Duration	What was the duration of the inter views or focus group?	Methods, Page 5
22. Data saturation	Was data saturation discussed?	N/A
23. Transcripts returned	Were transcripts returned to participants for comment and/or correction?	N/A
**Domain 3: analysis and findings**		
Data analysis		
24. Number of data coders	How many data coders coded the data?	Methods, Page 5
25. Description of the coding tree	Did authors provide a description of the coding tree?	Methods, Page 5 & Page 6 describes the coding process but no coding tree is given.
26. Derivation of themes	Were themes identified in advance or	Derived from the

For peer review only

	derived from the data?	data. Methods, Page 6
27. Software	What software, if applicable, was used to manage the data?	NVivo 11. Methods, Page 5
28. Participant checking	Did participants provide feedback on the findings?	N/A
Reporting		
29. Quotations presented	Were participant quotations presented to illustrate the themes/findings? Was each quotation identified? e.g. participant number	Results, Pages 6-10 All participants allocated a number.
30. Data and findings consistent	Was there consistency between the data presented and the findings?	Results, Pages 6-10 Discussion, pages 10-14.
31. Clarity of major themes	Were major themes clearly presented in the findings?	Results, Pages 6-10
32. Clarity of minor themes	Is there a description of diverse cases or discussion of minor themes?	Results, Pages 6-10 Sub-themes discussed. High level of agreement between participants. Strongly endorsed themes.

Once you have completed this checklist, please save a copy and upload it as part of your submission. When requested to do so as part of the upload process, please select the file type: *Checklist*. You will NOT be able to proceed with submission unless the checklist has been uploaded. Please DO NOT include this checklist as part of the main manuscript document. It must be uploaded as a separate file.

For peer review only

BMJ Open

Exploring psychiatrists' perspectives of working with patients with dissociative seizures in the UK healthcare system as part of the CODES trial – A qualitative study

Journal:	*BMJ Open*
Manuscript ID	bmjopen-2018-026493.R1
Article Type:	Research
Date Submitted by the Author:	11-Jan-2019
Complete List of Authors:	Jordan, Harriet; Institute of Psychiatry, Psychology, and Neuroscience, King's College London, De Crespigny Park, London, SE5 8AF, Psychology<br>Feehan, Sarah; Institute of Psychiatry, Psychology, and Neuroscience, King's College London, De Crespigny Park, London, SE5 8AF, Psychology<br>Perdue, Iain; Institute of Psychiatry, Psychology, and Neuroscience, King's College London, De Crespigny Park, London, SE5 8AF, Psychology<br>Murray, Joanna; Institute of Psychiatry, Psychology and Neuroscience, King's College London, Health Service and Population Research<br>Goldstein, Laura; Institute of Psychiatry, Psychology, and Neuroscience, King's College London, De Crespigny Park, London, SE5 8AF, Psychology
**Primary Subject Heading**:	Mental health
Secondary Subject Heading:	Qualitative research
Keywords:	Dissociative seizures, psychiatrists, qualitative

SCHOLARONE™
Manuscripts

Revision

Exploring psychiatrists' perspectives of working with patients with dissociative seizures in the UK healthcare system as part of the CODES trial – A qualitative study

Harriet Jordan MSc [1], Sarah Feehan BSc [1], Iain Perdue MPhil [1], Joanna Murray BA[2] & Laura H. Goldstein PhD FBPsS [1]

[1] King's College London, Department of Psychology, Institute of Psychiatry, Psychology and Neuroscience, London, UK.

[2] King's College London, Department of Health Service and Population Research, Institute of Psychiatry, Psychology and Neuroscience, London, UK.

Corresponding author: Prof Laura H. Goldstein, Department of Psychology, Institute of Psychiatry, Psychology, and Neuroscience, King's College London, PO77, De Crespigny Park, London, SE5 8AF, UK. Tel ++44 (0)20 7848 0218. laura.goldstein@kcl.ac.uk

Orcid ID:
Harriet Jordan https://orcid.org/0000-0002-7268-7310
Sarah Feehan https://orcid.org/0000-0003-3986-2698
Iain Perdue https://orcid.org/0000-0002-8078-8146
Joanna Murray https://orcid.org/0000-0002-7348-7505
Laura H Goldstein https://orcid.org/0000-0001-9387-3035

Abstract 298 words
Text word count: 4365

Key words: Dissociative seizures; psychiatrists; qualitative.

I, the Submitting Author has the right to grant and does grant on behalf of all authors of the Work (as defined in the below author licence), an exclusive licence and/or a non-exclusive licence for contributions from authors who are: i) UK Crown employees; ii) where BMJ has agreed a CC-BY licence shall apply, and/or iii) in accordance with the terms applicable for US Federal Government officers or employees acting as part of their official duties; on a worldwide, perpetual, irrevocable, royalty-free basis to BMJ Publishing Group Ltd ("BMJ") its licensees and where the relevant Journal is co-owned by BMJ to the co-owners of the Journal, to publish the Work in BMJ Open and any other BMJ products and to exploit all rights, as set out in our licence.

For peer review only - http://bmjopen.bmj.com/site/about/guidelines.xhtml

ABSTRACT

Objective: There is currently limited research exploring healthcare professionals' (HCPs) experiences of working with patients with dissociative seizures (DS). Existing studies do not focus on the role of psychiatrists in treating this complex condition. The objective of this study was to gain an understanding of United Kingdom (UK) based psychiatrists' experiences of the DS patient group. Against the backdrop of a UK-wide randomised controlled trial (RCT), focus was broadened to encompass issues arising in everyday practice with the DS patient group.

Design, Participants and Methods: A qualitative study using semi-structured interviews was undertaken with 10 psychiatrists currently working with DS patients within the context of a large RCT investigating treatments for DS. Thematic analysis was used to identify key themes and subthemes.

Setting: The psychiatrists were working in Liaison or Neuropsychiatry services in England.

Results: The key themes identified were other HCPs' attitudes to DS and the challenges of the DS patient group. There is a clear knowledge gap regarding DS for many HCPs and other clinical services can be reluctant to take referrals for this patient group. Important challenges posed by this patient group included avoidance (of difficult emotions and help), alexithymia and interpersonal difficulties. Difficulties with alexithymia meant DS patients could struggle to identify triggers for their seizures and to express their emotions. Interpersonal difficulties raised included difficulties in attachment with both HCPs and family members.

Conclusions: A knowledge gap for HCPs regarding DS has been identified and needs to be addressed to improve patient care. Given the complexity of the patient group and that clinicians from multiple disciplines will come into contact with DS patients, it is essential for any educational strategy to be implemented across the whole range of specialties, and to account for those already in practise as well as future trainees.

Article summary:

Strengths and limitations of this study

- This study uniquely explores the experiences of psychiatrists providing healthcare to patients with dissociative seizures.
- The findings have implications for guidance on interventions for people with dissociative seizures, specifically in relation to epilepsy.

For peer review only - http://bmjopen.bmj.com/site/about/guidelines.xhtml

- The study has a small sample size of 10 psychiatrists. The psychiatrists were all currently working at healthcare centres across England.

- Psychiatrists working with DS patients in Scotland and Wales are not part of our sample.

For peer review only - http://bmjopen.bmj.com/site/about/guidelines.xhtml

BACKGROUND

Dissociative seizures (DS) (often also referred to as Psychogenic Nonepileptic Seizures (PNES), Non-Epileptic Attack Disorder (NEAD), or functional seizures) are similar in appearance to epileptic seizures without the abnormal neural activity. The incidence of DS is reported as approximately 4.9 per 100,000/per year, [1] with some estimates reaching as high as 50 per 100,000/per year.[2] DS are a common challenge in epilepsy centres worldwide, [3 4] with between 12 and 20% of patients referred for telemetry having coexisting or misdiagnosed DS. [5 6]

Quantitative research has indicated that there is a gap in the knowledge of healthcare professionals (HCPs) regarding DS[7] and that some HCPs have a negative attitude towards patients with DS, perceiving the seizures as being under their control and seeing DS as untreatable. [8 9 10] Patients often describe feeling hopeless [11] and negative experiences with HCPs are frequently reported. [12] Previous research has found that clinicians, including general practitioners (GPs), have felt uncertainty in treating patients with DS [13 14] due to the lack of substantial evidence-base for any one particular intervention. Similar results have also been found in the Danish pediatric setting, where clinicians also reported a lack of sufficient treatment options and a need for clinical guidance (Nielsen et al., 2018)[15], further demonstrating the impact of DS across age groups and cultures.

The CODES (COgnitive behavioural therapy vs standardised medical care for adults with Dissociative non-Epileptic Seizures) Trial is the first sufficiently powered multi-centre, pragmatic, parallel group randomised controlled trial (RCT) to investigate the effectiveness of any psychological therapy for patients with DS. CODES is evaluating the clinical effectiveness and cost-effectiveness of cognitive behavioural therapy (CBT) plus trial standardised medical care (SMC) compared with SMC alone. [16] Each patient recruited into the study was first seen by a neurologist and then referred on to a psychiatrist for assessment. This care pathway was not always normally available outside of the CODES Trial in some areas of the United Kingdom (UK) within the context of the National Health Service (NHS).

When evaluating complex interventions such as those tested in the CODES Trial, it can be difficult to capture effectiveness using only quantitative methods.[17] Within CODES, data on clinicians' views of the DS patient group and the intervention was felt to be most appropriately captured using qualitative methodology as it would allow participants to elaborate on their responses rather than being constrained by questionnaires. Research has found that combining quantitative and qualitative methods within a study overall provides essential insight into how and why an intervention is effective,

For peer review only - http://bmjopen.bmj.com/site/about/guidelines.xhtml

if at all [18 19]. The purpose of this study was to gain an understanding of attitudes and beliefs among psychiatrists who had been part of the CODES Trial and were experienced in working with patients with DS, with particular emphasis on psychiatrists' views of other HCPs' ability and willingness to work with DS patients in the context of the NHS.

For peer review only

For peer review only - http://bmjopen.bmj.com/site/about/guidelines.xhtml

METHODS

Study population

Ten participants were purposively selected from the 29 psychiatrists involved in the CODES RCT to encompass the geographical distribution of the CODES Trial and the range of experience treating functional neurological disorders (FNDs), particularly DS. All participants were known to the wider CODES team prior to taking part in this qualitative study. CODES Trial grant holders were excluded to avoid study design-related bias. The psychiatrists were based at nine different NHS Trust tertiary or secondary mental health services across England with one based in a specialist neurological hospital. Recruitment took place between June and September 2017.

Data Collection

Those selected were initially contacted via email by HJ and invited to take part. Half of those approached had a prior working relationship with HJ within the CODES Trial. They were provided with an information sheet and a description of the aims of the project. If they were interested, a work place based face-to-face interview was scheduled at a time and date convenient for them. All participants provided informed consent to be interviewed. There were no refusals to participate. All interviews were conducted by HJ and recorded using an encrypted digital voice recorder to ensure data security and confidentiality. Interviews lasted between 41 – 96 minutes, covering the complete interview schedule.

Due to the nature of responses, it was not possible to determine the duration of responses solely covering the themes discussed in the current paper.

Interview Schedule

The interview schedule was developed by members of the CODES study team of which all authors were a part. The topics covered experiences of delivering the CODES SMC and involvement in the CODES RCT more generally (which will be reported elsewhere). In addition, topics covered the delivery of diagnosis, DS in the context of the NHS, and the challenges of the patient group, which are the focus of this paper. Participants were encouraged to give examples where possible and probing techniques were used to explore responses and elicit further detail where necessary [20] (see Supplementary file). The interview began with a series of questions about aspects of the trial processes which will be reported elsewhere. Although the topic guide focussed on involvement in the CODES trial and distinguished this way of working from more general issues of working with patients with DS, the

For peer review only - http://bmjopen.bmj.com/site/about/guidelines.xhtml

nature of responses meant that these topics often overlapped, and participants sometimes discussed issues relevant to multiple topics in a single answer. Participants readily elaborated with examples from their practise and experience outside of their involvement in the CODES RCT and it became apparent during data analysis that the themes covered warranted the focus of the current paper.

Data Analysis

The interviews were transcribed verbatim by members of the CODES research team. During the transcription process the interview data was anonymised. Completed transcripts were checked by HJ against the original recordings to ensure accuracy. The semi-structured interviews were analysed using thematic analysis. [21] This method was chosen rather than, for example, grounded theory, as our aim was to understand participants' professional views and methods of working with patients with DS rather than to develop theory. Three of the completed transcripts were chosen at random and coded initially by HJ, SF and another member of the research team. Emerging findings and preliminary themes were discussed in team meetings. HJ and SF then coded all 10 transcripts independently, using the qualitative data analysis software NVivo 11 (QSR International). NVivo allows the researcher to see how many interviews referred to a particular theme. Coding was done independently to allow for an organic and reflexive process. All content was grouped into categories to allow for the identification of patterns in the data. As each interview was analysed, new categories were added to the list and content was organized under each relevant category. Regular meetings were held to discuss agreements in coding and establish the parameters of each major theme. Major themes were established from the categories that contained the most substantial amount of data. Themes that had been identified by both coders were then combined, with subthemes being organized under the appropriate over-arching theme. We believe saturation had been reached since, as the interviews progressed, it was clear no new major themes were being elicited.

Patient and public involvement

The CODES Trial has a number of service users (i.e. individuals with DS or other relevant conditions) involved as members of its management committees, contributing to decisions about the running of the study and commenting on project outputs (e.g. papers).

RESULTS

Interviews from the 10 psychiatrists were analysed (see Table 1 for the psychiatrists' demographic characteristics). In general, there was a consistent level of agreement among participants on most

For peer review only

For peer review only - http://bmjopen.bmj.com/site/about/guidelines.xhtml

topics covered. This made it straightforward to identify main themes and clearly convey the conclusions drawn from the clinicians. Though the topic guide elicited a broad range of themes, for the purposes of this paper we focused on those that had significant clinical implications. Other themes relating to the CODES Trial will be described elsewhere. Two main clinically relevant overarching themes emerged from the data: i) Other Healthcare Professionals and DS and (ii) Psychiatrists' Identified Challenges of Working with DS.

Table 1 *Psychiatrists' self-reported demographic information*

	N	%		N	%
**Age**			**Sub Specialist Accreditation**		
31-40	1	10%	Liaison Psychiatry	6	60%
41-50	8	80%	Neuropsychiatry	1	10%
51-60	1	10%	Both	3	30%
**Gender**			**Years of Experience**		
Female	5	50%	11-15	5	50%
Male	5	50%	16-20	2	20%
			21-25	1	10%
**Location**			26-30	2	20%
London	6	60%			
Rest of England	4	40%			

Other Healthcare Professionals (HCPs) and DS

HCPs ill-equipped to deal with DS

Psychiatrists reported that HCPs from other services often felt uncertain when dealing with DS patients or were not prepared to work with patients with functional neurological symptoms. Others felt that DS is a disorder that GPs should better understand. It was also reported that services would often contribute to the diagnostic confusion by continually mistaking DS for epilepsy, despite referrals stating otherwise;

> *"They would come back saying well, look, this is epilepsy, they need to be seeing a neurologist, or people would end up back on anticonvulsants"*
> *(Psychiatrist 09, Female, Liaison Psychiatry).*

For peer review only - http://bmjopen.bmj.com/site/about/guidelines.xhtml

The mention of seizures would often result in a panicked response from some primary care psychology services that meant patients could sense having something difficult to treat. Psychiatrists described patients often feeling other clinicians had not given a positive message about a DS diagnosis, with some GPs reportedly stating the need to be on an anticonvulsant simply at the mention of seizures. This continual reference to epilepsy by other professionals can have a negative impact on patients' progress,

> *"They have said, oh this sounds…you have epilepsy. I say don't say that, you're not qualified to say that, you know, you do your job, uh because that one word would put patients (pause) back, by a year or two or ten sessions."*
> *(Psychiatrist 06, Male, Neuropsychiatry)*

Psychiatrists would find that making DS referrals to psychology services would result in the referral being rejected unless patients had a comorbidity that psychologists felt they could treat;

> *"So, if you send a referral saying this person has dissociative seizures, will you see them, they will return the referral, so you have to say, "this person has dissociative seizures; however, they also have a very clear anxiety or panic disorder and that is what I would like you to work on" and then they will accept it."*
> *(Psychiatrist 03, Female, Liaison Psychiatry)*

This was reiterated throughout most of the interviews, with psychiatrists stating that local services would prefer to treat comorbidities rather than the DS themselves and where no comorbidity could be identified, services often rejected the referrals. The majority of the interviewees endorsed the view that psychiatrists were a key part of DS patient care. However, two of the 10 questioned whether it was necessary in all cases for a psychiatrist to be involved especially if the DS patient had no clear psychiatric co-morbidities. This approach seemed to be influenced both by their usual practice and service pressures at the two trusts.

The need for experience

One conclusion frequently drawn from psychiatrists' experiences with HCPs in other services was that, in order to diagnose and treat DS, the clinician needed to have a significant level of experience with the disorder and that treatment should be undertaken in a specialist setting,

For peer review only - http://bmjopen.bmj.com/site/about/guidelines.xhtml

> *"I sincerely believe that…it's not a condition which anybody or everybody can deal with and I don't think it should be dealt with at IAPT level"*

(Psychiatrist 06, Male, Neuropsychiatry). (IAPT = Improving Access to Psychological Therapies services; the IAPT programme began in 2008 and aims to offer short-term evidence based psychological treatments for depression and anxiety in adults across England).

Delivering treatment in a specialist centre was described as not only helpful in terms of clinicians knowing how to work with DS, but would provide reassurance for patients that they were being seen by someone who is confident and experienced;

> *"I think it's one of those conditions where seeing people who know what it is, know what to do with it even if they can't promise to get it better it reduces everybody's anxiety levels about it" (Psychiatrist 09, Female, Liaison Psychiatry).*

This sense of needing experience was also reported as helpful in enabling professionals to acknowledge the amount that can often be unknown about the causes and triggers for DS and for helping the patient embrace that as well.

Psychiatrists' Identified Challenges of Working with DS

Avoidance

Avoidance was viewed as a key area of difficulty for the DS patient group and was noted to take a number of forms across 9/10 interviews. Examples of avoidance were given in the interviews but these fall into two broad categories: "avoidance of help" and "avoidance of emotions". Avoidance of help included not reading information about DS even when handed this directly in an appointment and avoiding attending medical or therapy appointments. Avoidance of emotions included a desire to take medication rather than deal with difficult feelings and blocking emotions;

> *"Quite a lot of people may have blocked out so to speak, the more emotional side of things and try to get on with things…. if they start to go through a more open exploration of the issues this can be very emotionally distressing and suddenly their mood goes down…… a lot of patients will have to go through that turbulence in order to come out on the other end having felt the issues, recognised them and dealt with it"*
>
> (Psychiatrist 06, Male, Neuropsychiatry)

For peer review only - http://bmjopen.bmj.com/site/about/guidelines.xhtml

The seizures themselves were seen as potentially fitting into a pattern of avoidant behaviour as a defence against difficult emotions. Avoidant behaviour could present under the guise of other difficulties such as a reluctance to travel to appointments.

Linked to the theme of avoidance was emotional literacy. This was commented on by 9/10 interviewees as a key difficulty for the DS patient group. It follows that if a person lacks awareness of their own emotions they would struggle to express these to others. This lack of emotional awareness could then impede the ability to make links between life events and feelings whilst in treatment.

> *"No symptoms, happy go lucky kind of personality, I love my family, no trauma, no pain and those people are the hardest"*
> (Psychiatrist 10, Female, Liaison Psychiatry)

Some of the most challenging of the DS patient group were those for whom no trigger for the seizures could be identified. Sometimes even when it was very clear to the clinician that there was a current stressor (such as caring for a gravely ill partner) patients with DS might deny this was the case. This seemed to lead to feelings of frustration for the psychiatrists as they viewed patients with no identified psychological trigger harder to treat successfully.

Complex interpersonal relationships

Eight out of the 10 psychiatrists noted that a patient with DS may well struggle with relationships, both with people generally and within the clinician-patient relationship. This could be associated with difficulties in attachment with the DS patient becoming over-attached and then not wanting to engage with any other clinician or be discharged.

> *"She became quite attached; there were real attachment issues…. so, I only managed to discharge her as I said a few weeks ago…… she didn't connect with that person [CODES CBT therapist] and she created a split between that person and me and she was like I just want to come and see you, can't I just come and see you every two weeks?"*
> (Psychiatrist 08, Female Liaison Psychiatry)

Attempts at splitting were described as occurring not only between individual clinicians (as above) but also between services e.g., a specialist national service being "good" and all local services being "bad". This splitting and idealisation of one service or clinician could be accompanied by unrealistic

For peer review only - http://bmjopen.bmj.com/site/about/guidelines.xhtml

expectations that the psychiatrist would continue to see them indefinitely. A partner being in the room throughout every appointment can mean the patient is less able to be open about how they are feeling and certainly less likely to discuss any current relationship difficulties. A co-dependent relationship may also impact on motivation to recover as some people with DS have social circumstances in which getting better may feel riskier than remaining unwell. A couple of the psychiatrists went further and conceptualised some examples of factitious behaviours in patients (e.g. deliberately concealing medication non-adherence from family and clinicians) as driven by a profound need to be looked after.

DISCUSSION

We present here the experiences and views of the psychiatrists involved in an RCT for patients with DS. The characteristics of the nested group were similar to the clinicians involved in the trial as a whole in relation to age, gender, ethnicity and experience working with DS. Participants generally expressed concordant views across the range of interview questions, suggesting that issues surrounding DS are very apparent to the professionals working closely with this population. Views broadly support previous research that describes DS patients as a heterogeneous population with complex presentations and demanding HCP input, [8 10 21] as well as the need for an improvement in education and awareness of DS by HCPs. [22]

Participants believed that interventions by other HCPs at times made their own work with this patient group more difficult. They identified a knowledge gap surrounding DS among other HCPs. Previous research has found that HCPs from a variety of backgrounds often have very different perceptions regarding DS, [23] which seems to be a pervasive problem at all levels of health services in the UK, from GPs and primary care services to Community Mental Health Trusts and Emergency Departments. While we cannot report here on the perceptions of other HCPs involved in the CODES Trial, what is perhaps most significant is the current participants' observation that this knowledge gap can at times have a detrimental effect on patients' prognosis, with one participant noting that the mention of epilepsy and AED treatment can set a patient's progress back significantly. Rawlings and Reuber's recent review[10] raised concerns not only about HCPs' DS knowledge gap but also negative attitudes towards the condition. It is of concern that negative attitudes towards the DS patient group may be created or reinforced by HCPs currently in practice when training junior staff. [10] Dworetzky [24] reported that epilepsy specialists in the USA when teaching junior staff about DS tend to focus on the cost of care and misuse of services caused by DS. Negative clinician attitudes towards DS patients themselves

For peer review only - http://bmjopen.bmj.com/site/about/guidelines.xhtml

For peer review only

did not emerge as a theme here, perhaps because our group of psychiatrists have chosen to work with this patient group.

Referring to epilepsy can be damaging to patients with DS as it contributes to diagnostic confusion in a number of ways. If they have been diagnosed with DS already, it may cause them to doubt whether the diagnosis is correct. It may initiate or strengthen a belief that they in fact have epilepsy, despite diagnostic evidence (e.g.; video-electroencephalography (vEEG) telemetry results) and clinical opinion to the contrary. [25] It also means that often DS patients are treated with potentially harmful anti-epileptic drugs (AEDs) with serious side effects despite them having no medical benefit and this can lead to serious iatrogenic harm. [26] It would be helpful for more specific epilepsy-related guidance to be developed for HCPs with regards to reducing AEDs and handling a misdiagnosis of epilepsy so as to avoid any setbacks in recovery. HCPs should be made aware of the clinical significance that simply mentioning epilepsy and AEDs can have on a patient and this should be highlighted in any future educational resources.

The International League Against Epilepsy (ILAE) PNES Task Force produced a special report describing the minimum requirements for a diagnosis of DS[27]. It would be beneficial for those working with DS to become familiar with these guidelines so that they can clearly convey their diagnostic reasoning to the patient and can discuss the features of DS and the characteristics distinguishing DS from epilepsy more confidently. In addition to this detailed account of the diagnosis, LaFrance et al. [25] also produced a comprehensive overview of the management of DS patients, encompassing diagnosis, treatment and maintaining engagement. Whilst it is argued that the management of DS requires expertise, the guidelines are accessible and can be used as a helpful tool for non-experts to familiarize themselves with the important elements for interacting with this patient group.

There were some discordant views surrounding which services and professionals should be able to work with DS. Some participants described the need for more specialist services to be made available and for psychiatric training to cover functional neurological symptoms in greater depth. However, some participants stressed the necessity for GPs and primary care clinicians to also become more familiar with DS. Despite this contrast, the consensus remains that more education and awareness are required for all HCPs regardless of their speciality. This supports previous research that has reported the need for more clinicians to be comfortable with treating all FNDs, particularly DS. [28] Encouragingly going forward even a brief training intervention for medical students and doctors' improved confidence levels and diagnostic accuracy when working with DS. [29 30]

For peer review only - http://bmjopen.bmj.com/site/about/guidelines.xhtml

To address this issue, better resources and educational materials need to be made available for those who are likely to be working with DS, both for future clinicians and clinicians currently in practice. Rommelfanger et al. [22] described a level of "professional isolation" often felt among care providers working with functional symptoms due to a distinct lack of formalised training and reported on the need for a shift in priorities to support clinicians working with patients with FNDs. This could start as early as medical school but should also involve the development of sufficient resources to support the multidisciplinary approach that is often required to treat FNDs.

Avoidant behaviours could be divided into two categories of "avoidance of help" and "avoidance of emotions". Linked to avoidance was the subtheme of emotional literacy, difficulty feeling and expressing emotions. These findings support previous quantitative research on alexithymia in patients with DS, where Bewley et al. [31] found DS patients had significantly greater difficulty identifying feelings than healthy controls. More recently, Uliaszek et al. [32] found significant emotion dysregulation among DS patients when compared with a control group. This may have important implications for future therapeutic developments, by incorporating elements of effective treatments for alexithymia from other treatment models (e.g. mindfulness-based therapy for alexithymia). [33] Previous research also found evidence of experiential avoidance [34] and avoidant coping styles [35] among the DS patient population. Given these findings, it is important to establish what impact this avoidant behaviour may have on treatment outcomes and what can be done to mitigate any negative impacts. Some participants in the present study reported positive progress in openly identifying the patients' avoidant behaviour and providing the opportunity to address it constructively. Treatment approaches for DS should make provision for tackling avoidance directly. [16 36] In addition, for a subset of the patient group, no trigger for DS is identified and these patients tended to be particularly difficult to treat from the point of explaining the diagnosis onwards. Working with this complex patient group effectively clearly needs experience and knowledge of the condition.

Our study has a number of limitations. The study has a small sample size of 10 psychiatrists currently working at healthcare centres around England. Psychiatrists working in Scotland and Wales are not represented in this group. As participants were all involved in the CODES Trial and knew the interviewer or other members of the research team, it is possible this influenced their responses. However, as the interview did not solely focus on the CODES Trial, other aspects may be less likely to have been affected by prior working relationships.

For peer review only - http://bmjopen.bmj.com/site/about/guidelines.xhtml

It is possible that the DS patient sample in the CODES Trial, who will have partly influenced the current psychiatrists' views, is not fully representative of the DS patient population in general. It is also likely that there is a self-selection bias for those presenting to psychiatrists as many DS patients may reject a psychiatric diagnosis and thus do not attend psychiatric appointments. [37] The disparity in services may mean that some patients simply do not have access to a specialist psychiatrist and are referred back to their GP. [38] We will report elsewhere an exploration of the views of patients with DS involved in the CODES Trial to triangulate findings and maximise our understanding of working and living with DS. All participants in this current study were specialist psychiatrists and therefore not representative of the population of psychiatrists more generally in the NHS across the UK. However, due to the organisation of care in the NHS, DS patients are not usually seen by general psychiatrists. Therefore, our sample is representative of those clinicians who are most likely to provide direct clinical contact with the DS patient population. It is possible that talking about the challenges of the patient group would have led the respondents to think more about their perception of the difficulties of working with this patient group and may have influenced the nature of the emerging themes.

In terms of strengths, to our knowledge, our study was the first qualitative study focusing solely on psychiatrists' perspectives of working with patients with DS. Prior published qualitative studies [39 40] interviewed healthcare professionals from a variety of backgrounds. However, McMillan et al.'s [39] large sample (74 interviews) only included epilepsy staff such as neurologists, EEG technicians and epilepsy nurses and no mental health care providers. In du Toit & Pretorius's [40] study, only three of 15 people interviewed were psychiatrists. Given that DS is classified as a mental health disorder (DSM5, [41]) and a dissociative disorder (ICD-10 [42]) and will predominantly be handled by mental health clinicians, it seems important to explore the views of those working with the DS population in the appropriate clinical context. In addition, it is likely that the current sample is more representative of the clinicians most likely to be working with DS in developed countries, and not limited to a military clinical environment. [39]

CONCLUSION

Qualitative findings suggest that patients with DS are a complex and at times challenging population that requires intervention from experienced clinicians familiar with the condition. Significantly, intervention can be made more difficult if not provided in an informed and experienced manner. Our findings have important implications for medical and allied professional training with regards to FNDs

For peer review only - http://bmjopen.bmj.com/site/about/guidelines.xhtml

in order for clinicians to be better equipped to recognise and handle the challenges that come with treating DS. It is also hoped that a greater evidence base for treatments for patients with DS will help eradicate the variability within healthcare provision.

Acknowledgements: The authors would like to thank the psychiatrists who were willing to be interviewed for this study given their busy schedules. The authors also thank Julie Read for her assistance with the project.

Author contributions: HJ, IP, JM and LHG contributed to the development of the topic guide. HJ carried out all data collection. HJ and SF transcribed the data, carried out the analysis and led the writing of the paper. JM and IP reviewed drafts of the paper. LHG oversaw the project, contributed to the writing of the paper and reviewed successive drafts. All authors approved the final version of the submitted text.

Funding statement: This paper describes independent research funded by the National Institute for Health Research (Health Technology Assessment programme, 12/26/01, COgnitive behavioural therapy vs standardised medical care for adults with Dissociative non-Epileptic Seizures: A multicentre randomised controlled trial (CODES)). LHG receives salary support from the National Institute for Health Research (NIHR) Maudsley Biomedical Research Centre at the South London and Maudsley NHS Foundation Trust and King's College London. The views expressed in this publication are those of the authors and not necessarily those of the NHS, the NIHR or the Department of Health.

Competing interests: LHG and JM report the grant from the NIHR HTA for the conduct of the study. None of the other authors have competing interests to declare.

Data sharing statement: Contact the corresponding author with queries.

Ethics approval: The study was approved by the NHS Health Research Authority, London – Camberwell St Giles Research Ethics Committee (reference number 13/LO/1595). Written informed consent was obtained from all study participants. Study registration: Current Controlled Trials: ISRCTN05681227; ClinicalTrials.gov: NCT02325544.

For peer review only - http://bmjopen.bmj.com/site/about/guidelines.xhtml

REFERENCES

1. Duncan R, Razvi S, Mulhern S. Newly presenting psychogenic nonepileptic seizures: Incidence, population characteristics, and early outcome from a prospective audit of a first seizure clinic. *Epilepsy Behav* 2011;20(2):308-11. doi: 10.1016/j.yebeh.2010.10.022
2. Kanemoto K, LaFrance WC, Duncan R, et al. PNES around the world: Where we are now and how we can close the diagnosis and treatment gaps—an ILAE PNES Task Force report. *Epilepsia Open* 2017;2(3):307-16. doi: doi:10.1002/epi4.12060
3. Asadi-Pooya AA, Sperling MR. Epidemiology of psychogenic nonepileptic seizures. *Epilepsy Behav* 2015;46:60-65. doi: https://doi.org/10.1016/j.yebeh.2015.03.015
4. Kerr MP, Mensah S, Besag F, et al. International consensus clinical practice statements for the treatment of neuropsychiatric conditions associated with epilepsy. *Epilepsia* 2011;52(11):2133-38. doi: 10.1111/j.1528-1167.2011.03276.x
5. Angus-Leppan H. Diagnosing epilepsy in neurology clinics: A prospective study. *Seizure* 2008;17(5):431-36. doi: 10.1016/j.seizure.2007.12.010
6. Lesser RP. Psychogenic seizures. *Neurology* 1996;46(6):1499-507.
7. Worsely C, Whitehead K, Kandler R, et al. Illness perceptions of health care workers in relation to epileptic and psychogenic nonepileptic seizures. *Epilepsy Behav* 2011;20(4):668-73. doi: https://doi.org/10.1016/j.yebeh.2011.01.029
8. Hingray C, El-Hage W, Duncan R, et al. Access to diagnostic and therapeutic facilities for psychogenic nonepileptic seizures: An international survey by the ILAE PNES Task Force. *Epilepsia* 2018;59(1):203-14. doi: doi:10.1111/epi.13952
9. Kanaan RA, Armstrong D, Wessely SC. Neurologists' understanding and management of conversion disorder. *J Neurol Neurosurg Psychiatry* 2011;82(9):961-6. doi: http://dx.doi.org/10.1136/jnnp.2010.233114
10. Rawlings GH, Reuber M. Health care practitioners' perceptions of psychogenic nonepileptic seizures: A systematic review of qualitative and quantitative studies. *Epilepsia* 2018;59(6):1109-23. doi: doi:10.1111/epi.14189
11. Karterud HN, Knizek BL, Nakken KO. Changing the diagnosis from epilepsy to PNES: Patients' experiences and understanding of their new diagnosis. *Seizure* 2010;19(1):40-46. doi: http://dx.doi.org/10.1016/j.seizure.2009.11.001
12. Rawlings GH, Reuber M. What patients say about living with psychogenic nonepileptic seizures: A systematic synthesis of qualitative studies. *Seizure* 2016;41:100-11. doi: http://dx.doi.org/10.1016/j.seizure.2016.07.014
13. Shneker BF, Elliott JO. Primary care and emergency physician attitudes and beliefs related to patients with psychogenic nonepileptic spells. *Epilepsy Behav* 2008;13(1):243-47. doi: https://doi.org/10.1016/j.yebeh.2008.03.001
14. O'Sullivan SS, Sweeney BJ, McNamara B. The opinion of the general practitioner toward clinical management of patients with psychogenic nonepileptic seizures. *Epilepsy Behav* 2006;8(1):256-60. doi: https://doi.org/10.1016/j.yebeh.2005.09.013
15. Nielsen ES, Wichaidit BT, Østergaard JR, et al. Paediatricians' attitudes to and management of functional seizures in children. *European Journal of Paediatric Neurology* 2018;22(5):774-81. doi: 10.1016/j.ejpn.2018.05.007
16. Goldstein LH, Mellers JDC, Landau S, et al. COgnitive behavioural therapy vs standardised medical care for adults with Dissociative non-Epileptic Seizures (CODES): A multicentre randomised controlled trial protocol. *BMC Neurology* 2015;15(1) doi: http://dx.doi.org/10.1186/s12883-015-0350-0
17. Lewin S, Glenton C, Oxman AD. Use of qualitative methods alongside randomised controlled trials of complex healthcare interventions: methodological study. *BMJ* 2009;339 doi: 10.1136/bmj.b3496

For peer review only - http://bmjopen.bmj.com/site/about/guidelines.xhtml

18. Möhler R, Köpke S, Meyer G. Criteria for Reporting the Development and Evaluation of Complex Interventions in healthcare: revised guideline (CReDECI 2). *Trials* 2015;16(1):204. doi: 10.1186/s13063-015-0709-y
19. Campbell M, Fitzpatrick R, Haines A, et al. Framework for design and evaluation of complex interventions to improve health. *BMJ* 2000;321 doi: 10.1136/bmj.321.7262.694
20. Rubin H, Rubin I. Qualitative interviewing: A method to the madness: Thousand Oaks, CA: Sage Publications, 1995.
21. Braun V, Clarke V. Using thematic analysis in psychology. *Qualitative Research in Psychology* 2006;3(2):77-101. doi: 10.1191/1478088706qp063oa
22. Rommelfanger KS, Factor SA, LaRoche S, et al. Disentangling Stigma from Functional Neurological Disorders: Conference Report and Roadmap for the Future. *Front Neurol* 2017;8(106) doi: 10.3389/fneur.2017.00106
23. Whitehead K, Reuber M. Illness perceptions of neurologists and psychiatrists in relation to epilepsy and nonepileptic attack disorder. *Seizure* 2012;21(2):104-9.
24. Dworetzky BA. What Are We Communicating When We Present the Diagnosis of PNES? *Epilepsy Curr* 2015;15(6):353-57. doi: 10.5698/1535-7511-15.6.353
25. Lafrance Jr WC, Reuber M, Goldstein LH. Management of psychogenic nonepileptic seizures. *Epilepsia* 2013;54:53-67. doi: http://dx.doi.org/10.1111/epi.12106
26. Reuber M, Elger CE. Psychogenic nonepileptic seizures: Review and update. *Epilepsy and Behavior* 2003;4(3):205-16.
27. Lafrance Jr WC, Baker GA, Duncan R, et al. Minimum requirements for the diagnosis of psychogenic nonepileptic seizures: A staged approach: A report from the International League Against Epilepsy Nonepileptic Seizures Task Force. *Epilepsia* 2013;54(11):2005-18. doi: http://dx.doi.org/10.1111/epi.12356
28. Gardiner P, MacGregor L, Carson A, et al. Occupational therapy for functional neurological disorders: a scoping review and agenda for research. *CNS Spectrums* 2017;23(3):205-12. doi: 10.1017/S1092852917000797 [published Online First: 11/27]
29. O'Sullivan SS, Redwood RI, Hunt D, et al. Recognition of psychogenic non-epileptic seizures: a curable neurophobia? *Journal of Neurology, Neurosurgery & Psychiatry* 2013;84(2):228-31. doi: 10.1136/jnnp-2012-303062
30. Seneviratne U, Ding C, Bower S, et al. Video-based training improves the accuracy of seizure diagnosis. *Journal of Neurology, Neurosurgery & Psychiatry* 2014 doi: 10.1136/jnnp-2013-306618
31. Bewley J, Murphy PN, Mallows J, et al. Does alexithymia differentiate between patients with nonepileptic seizures, patients with epilepsy, and nonpatient controls? *Epilepsy and Behavior* 2005;7(3):430-37.
32. Uliaszek AA, Prensky E, Baslet G. Emotion regulation profiles in psychogenic non-epileptic seizures. *Epilepsy Behav* 2012;23(3):364-69. doi: https://doi.org/10.1016/j.yebeh.2012.01.009
33. Norman H, Marzano L, Coulson M, et al. Effects of mindfulness-based interventions on alexithymia: a systematic review. *Evidence Based Mental Health* 2018
34. Dimaro LV, Dawson DL, Roberts NA, et al. Anxiety and avoidance in psychogenic nonepileptic seizures: the role of implicit and explicit anxiety. *Epilepsy & Behavior* 2014;33:77-86. doi: http://dx.doi.org/10.1016/j.yebeh.2014.02.016
35. Goldstein LH, Drew C, Mellers J, et al. Dissociation, hypnotizability, coping styles and health locus of control: Characteristics of pseudoseizure patients. *Seizure* 2000;9(5):314-22.
36. Goldstein LH, Chalder T, Chigwedere C, et al. Cognitive-behavioral therapy for psychogenic nonepileptic seizures A pilot RCT. *Neurology* 2010;74(24):1986-94.
37. Carton S, Thompson PJ, Duncan JS. Non-epileptic seizures: patients' understanding and reaction to the diagnosis and impact on outcome. *Seizure* 2003;12(5):287-94.

For peer review only - http://bmjopen.bmj.com/site/about/guidelines.xhtml

38. Agrawal N, Fleminger S, Ring H, et al. Neuropsychiatry in the UK: National survey of existing service provision. *Psychiatric Bulletin* 2008;32(8):288-91. doi: http://dx.doi.org/10.1192/pb.bp.107.018424
39. McMillan KK, Pugh MJ, Hamid H, et al. Providers' perspectives on treating psychogenic nonepileptic seizures: Frustration and hope. *Epilepsy and Behavior* 2014;37:276-81. doi: http://dx.doi.org/10.1016/j.yebeh.2014.07.001
40. du Toit A, Pretorius C. Psychogenic nonepileptic seizures: Namibian healthcare providers' perceptions and frustrations. *Seizure* 2017;50:43-52. doi: https://doi.org/10.1016/j.seizure.2017.06.004
41. American Psychiatric Association. Diagnostic and statistical manual of mental disorders Fifth edition. United States: American Psychiatric Association; 2013.
42. World Health Organization. The ICD-10 Classification of Mental and Behavioural Disorders: Clinical Descriptions and Diagnostic Guidelines: World Health Organization 1992.

For peer review only

For peer review only - http://bmjopen.bmj.com/site/about/guidelines.xhtml

Supplementary file

Psychiatrists' interview schedule from which current themes were elicited (material related to the conduct of the CODES Trial to be reported elsewhere)

Psychiatrists Interview Schedule

Section 1: Background and Specific Issues relating to the CODES RCT

How does CODES standardised medical care (SMC) differ from the techniques you would usually use to treat patients with DS?

If SMC is shown to reduce seizures, what do you think will be most difficult about making SMC standard across services?

Were there any parts of the CODES SMC approach that proved more challenging?

How did you manage significant deterioration in a participant's mental health during their time in the CODES study?

How did you feel about NOT referring to other types of therapy whilst a participant was in CODES?

Section 2: Experience of the Intervention

Did the way patients engaged with standardised medical care (SMC) seem to change over time?

Were there any 'lightbulb moments' in the course of SMC where patients appeared to have a sudden understanding of their condition?

Section 3: Individual Psychological, Social or Health-Related Differences and Impact on Treatment

Do you think that there were any factors that may have affected patients understanding of their diagnosis?

Were there any patients who may have been more suitable than others to receive SMC alone? If so, what distinguished these types of clients?

Were there issues that you had to address in order to improve engagement? Or were there any barriers to patients engaging with SMC?

For peer review only

Could sessions ever become side-tracked/derailed by other issues? E.g.: social issues, safeguarding or health-related concerns?

For peer review only

Consolidated criteria for reporting qualitative studies (COREQ): 32-item checklist

Developed from:
Tong A, Sainsbury P, Craig J. Consolidated criteria for reporting qualitative research (COREQ): a 32-item checklist for interviews and focus groups. *International Journal for Quality in Health Care*. 2007. Volume 19, Number 6: pp. 349 – 357

YOU MUST PROVIDE A RESPONSE FOR ALL ITEMS. ENTER N/A IF NOT APPLICABLE

**No. Item**	**Guide questions/description**	**Reported on Page #**
**Domain 1: Research team and reflexivity**		
Personal Characteristics		
1. Inter viewer/facilitator	Which author/s conducted the **interviews** or focus group?	Methods, Page 5
2. Credentials	What were the researcher's credentials? E.g. PhD, MD	Authors, Page 1
3. Occupation	What was their occupation at the time of the study?	Methods, Page 5, where we state that The interview schedule was developed by members of the CODES study team, of which all authors were members.
4. Gender	Was the researcher male or female?	Authors list. Page 1
5. Experience and training	What experience or training did the researcher have?	Author JM is a qualitative research expert and supervised the project.
Relationship with participants		
6. Relationship established	Was a relationship established prior to study commencement?	Member of the CODES study research team had prior involvement with participants, as all interviewees are involved in this study. Participants, Page 5.
7. Participant knowledge of the interviewer	What did the participants know about the researcher? e.g. personal goals, reasons for doing the research	See above.
8. Interviewer characteristics	What characteristics were reported about the inter viewer/facilitator? e.g. Bias, assumptions, reasons and interests in the	N/A We have not reported this as we

For peer review only - http://bmjopen.bmj.com/site/about/guidelines.xhtml

	research topic	are not undertaking IPA.
**Domain 2: study design**		
Theoretical framework		
9. Methodological orientation and Theory	What methodological orientation was stated to underpin the study? e.g. grounded theory, discourse analysis, ethnography, phenomenology, content analysis	Thematic Analysis. Methods, Page 5.
Participant selection		
10. Sampling	How were participants selected? e.g. **purposive**, convenience, consecutive, snowball	Methods, Page 5.
11. Method of approach	How were participants approached? e.g. face-to-face, telephone, mail, **email**	Methods, Page 5
12. Sample size	How many participants were in the study?	Methods, Page 5
13. Non-participation	How many people refused to participate or dropped out? Reasons?	No refusals. N/A.
Setting		
14. Setting of data collection	Where was the data collected? e.g. home, clinic, workplace	Face to face. Methods, page 5
15. Presence of non-participants	Was anyone else present besides the participants and researchers?	N/A
16. Description of sample	What are the important characteristics of the sample? e.g. demographic data, date	Results, Demographic Table Page 6
Data collection		
17. Interview guide	Were questions, prompts, guides provided by the authors? Was it pilot tested?	Methods, page 5 Prompts & Probes used. Not pilot tested.
18. Repeat interviews	Were repeat inter views carried out? If yes, how many?	No repeat interviews conducted. N/A.
19. Audio/visual recording	Did the research use audio or visual recording to collect the data?	Methods, Page 5
20. Field notes	Were field notes made during and/or after the inter view or focus group?	N/A
21. Duration	What was the duration of the inter views or focus group?	Methods, Page 5
22. Data saturation	Was data saturation discussed?	Yes at the end of the Data Analysis section
23. Transcripts returned	Were transcripts returned to participants for comment and/or correction?	N/A
**Domain 3: analysis and findings**		
Data analysis		
24. Number of data coders	How many data coders coded the data?	Methods, Page 5
25. Description of the coding tree	Did authors provide a description of the coding tree?	Methods, Page 5 & Page 6 describes the coding process

For peer review only

		but no coding tree is given.
26. Derivation of themes	Were themes identified in advance or derived from the data?	Derived from the data. Methods, Page 6
27. Software	What software, if applicable, was used to manage the data?	NVivo 11. Methods, Page 5
28. Participant checking	Did participants provide feedback on the findings?	N/A
Reporting		
29. Quotations presented	Were participant quotations presented to illustrate the themes/findings? Was each quotation identified? e.g. participant number	Results, Pages 6-10 All participants allocated a number.
30. Data and findings consistent	Was there consistency between the data presented and the findings?	Results, Pages 6-10 Discussion, pages 10-14.
31. Clarity of major themes	Were major themes clearly presented in the findings?	Results, Pages 6-10
32. Clarity of minor themes	Is there a description of diverse cases or discussion of minor themes?	Results, Pages 6-10 Sub-themes discussed. High level of agreement between participants. Strongly endorsed themes.

Once you have completed this checklist, please save a copy and upload it as part of your submission. When requested to do so as part of the upload process, please select the file type: *Checklist*. You will NOT be able to proceed with submission unless the checklist has been uploaded. Please DO NOT include this checklist as part of the main manuscript document. It must be uploaded as a separate file.

For peer review only

BMJ Open

Exploring psychiatrists' perspectives of working with patients with dissociative seizures in the UK healthcare system as part of the CODES trial – A qualitative study

Journal:	*BMJ Open*
Manuscript ID	bmjopen-2018-026493.R2
Article Type:	Research
Date Submitted by the Author:	08-Feb-2019
Complete List of Authors:	Jordan, Harriet; Institute of Psychiatry, Psychology, and Neuroscience, King's College London, De Crespigny Park, London, SE5 8AF, Psychology<br>Feehan, Sarah; Institute of Psychiatry, Psychology, and Neuroscience, King's College London, De Crespigny Park, London, SE5 8AF, Psychology<br>Perdue, Iain; Institute of Psychiatry, Psychology, and Neuroscience, King's College London, De Crespigny Park, London, SE5 8AF, Psychology<br>Murray, Joanna; Institute of Psychiatry, Psychology and Neuroscience, King's College London, Health Service and Population Research<br>Goldstein, Laura; Institute of Psychiatry, Psychology, and Neuroscience, King's College London, De Crespigny Park, London, SE5 8AF, Psychology
**Primary Subject Heading**:	Mental health
Secondary Subject Heading:	Qualitative research
Keywords:	Dissociative seizures, psychiatrists, qualitative

SCHOLARONE™
Manuscripts

2nd Revision

Exploring psychiatrists' perspectives of working with patients with dissociative seizures in the UK healthcare system as part of the CODES trial – A qualitative study

Harriet Jordan MSc [1], Sarah Feehan BSc [1], Iain Perdue MPhil [1], Joanna Murray BA[2] & Laura H. Goldstein PhD FBPsS [1]

[1] King's College London, Department of Psychology, Institute of Psychiatry, Psychology and Neuroscience, London, UK.

[2] King's College London, Department of Health Service and Population Research, Institute of Psychiatry, Psychology and Neuroscience, London, UK.

Corresponding author: Prof Laura H. Goldstein, Department of Psychology, Institute of Psychiatry, Psychology, and Neuroscience, King's College London, PO77, De Crespigny Park, London, SE5 8AF, UK. Tel ++44 (0)20 7848 0218. laura.goldstein@kcl.ac.uk

Orcid ID:
Harriet Jordan https://orcid.org/0000-0002-7268-7310
Sarah Feehan https://orcid.org/0000-0003-3986-2698
Iain Perdue https://orcid.org/0000-0002-8078-8146
Joanna Murray https://orcid.org/0000-0002-7348-7505
Laura H Goldstein https://orcid.org/0000-0001-9387-3035

Abstract 298 words
Text word count: 4405

Key words: Dissociative seizures; psychiatrists; qualitative.

I, the Submitting Author has the right to grant and does grant on behalf of all authors of the Work (as defined in the below author licence), an exclusive licence and/or a non-exclusive licence for contributions from authors who are: i) UK Crown employees; ii) where BMJ has agreed a CC-BY licence shall apply, and/or iii) in accordance with the terms applicable for US Federal Government officers or employees acting as part of their official duties; on a worldwide, perpetual, irrevocable, royalty-free basis to BMJ Publishing Group Ltd ("BMJ") its licensees and where the relevant Journal is co-owned by BMJ to the co-owners of the Journal, to publish the Work in BMJ Open and any other BMJ products and to exploit all rights, as set out in our licence.

For peer review only

For peer review only - http://bmjopen.bmj.com/site/about/guidelines.xhtml

ABSTRACT

Objective: There is currently limited research exploring healthcare professionals' (HCPs) experiences of working with patients with dissociative seizures (DS). Existing studies do not focus on the role of psychiatrists in treating this complex condition. The objective of this study was to gain an understanding of United Kingdom (UK) based psychiatrists' experiences of the DS patient group. Against the backdrop of a UK-wide randomised controlled trial (RCT), focus was broadened to encompass issues arising in everyday practice with the DS patient group.

Design, Participants and Methods: A qualitative study using semi-structured interviews was undertaken with 10 psychiatrists currently working with DS patients within the context of a large RCT investigating treatments for DS. Thematic analysis was used to identify key themes and subthemes.

Setting: The psychiatrists were working in Liaison or Neuropsychiatry services in England.

Results: The key themes identified were other HCPs' attitudes to DS and the challenges of the DS patient group. There is a clear knowledge gap regarding DS for many HCPs and other clinical services can be reluctant to take referrals for this patient group. Important challenges posed by this patient group included avoidance (of difficult emotions and help), alexithymia and interpersonal difficulties. Difficulties with alexithymia meant DS patients could struggle to identify triggers for their seizures and to express their emotions. Interpersonal difficulties raised included difficulties in attachment with both HCPs and family members.

Conclusions: A knowledge gap for HCPs regarding DS has been identified and needs to be addressed to improve patient care. Given the complexity of the patient group and that clinicians from multiple disciplines will come into contact with DS patients, it is essential for any educational strategy to be implemented across the whole range of specialties, and to account for those already in practise as well as future trainees.

Article summary:

Strengths and limitations of this study

- This study uniquely explores the experiences of psychiatrists providing healthcare to patients with dissociative seizures (DS).
- The findings have implications for guidance on interventions for people with dissociative seizures, specifically in relation to epilepsy.

For peer review only - http://bmjopen.bmj.com/site/about/guidelines.xhtml

- The study has a small sample size of 10 psychiatrists. The psychiatrists were all currently working at healthcare centres across England.
- Psychiatrists working with DS patients in Scotland and Wales are not part of our sample.
- All the participants interviewed in this study were specialist psychiatrists with an interest and experience in working with patients with DS and therefore not representative of the population of psychiatrists more generally in the National Health Service across the UK.

For peer review only

For peer review only - http://bmjopen.bmj.com/site/about/guidelines.xhtml

BACKGROUND

Dissociative seizures (DS) (often also referred to as Psychogenic Nonepileptic Seizures (PNES), Non-Epileptic Attack Disorder (NEAD), or functional seizures) are similar in appearance to epileptic seizures without the abnormal neural activity. The incidence of DS is reported as approximately 4.9 per 100,000/per year, [1] with some estimates reaching as high as 50 per 100,000/per year.[2] DS are a common challenge in epilepsy centres worldwide, [3 4] with between 12 and 20% of patients referred for telemetry having coexisting or misdiagnosed DS. [5 6]

Quantitative research has indicated that there is a gap in the knowledge of healthcare professionals (HCPs) regarding DS[7] and that some HCPs have a negative attitude towards patients with DS, perceiving the seizures as being under their control and seeing DS as untreatable. [8 9 10] Patients often describe feeling hopeless [11] and negative experiences with HCPs are frequently reported. [12] Previous research has found that clinicians, including general practitioners (GPs), have felt uncertainty in treating patients with DS [13 14] due to the lack of substantial evidence-base for any one particular intervention. Similar results have also been found in the Danish pediatric setting, where clinicians also reported a lack of sufficient treatment options and a need for clinical guidance (Nielsen et al., 2018)[15], further demonstrating the impact of DS across age groups and cultures.

The CODES (COgnitive behavioural therapy vs standardised medical care for adults with Dissociative non-Epileptic Seizures) Trial is the first sufficiently powered multi-centre, pragmatic, parallel group randomised controlled trial (RCT) to investigate the effectiveness of any psychological therapy for patients with DS. CODES is evaluating the clinical effectiveness and cost-effectiveness of cognitive behavioural therapy (CBT) plus trial standardised medical care (SMC) compared with SMC alone. [16] Each patient recruited into the study was first seen by a neurologist and then referred on to a psychiatrist for assessment. This care pathway was not always normally available outside of the CODES Trial in some areas of the United Kingdom (UK) within the context of the National Health Service (NHS).

When evaluating complex interventions such as those tested in the CODES Trial, it can be difficult to capture effectiveness using only quantitative methods.[17] Within CODES, data on clinicians' views of the DS patient group and the intervention was felt to be most appropriately captured using qualitative methodology as it would allow participants to elaborate on their responses rather than being constrained by questionnaires. Research has found that combining quantitative and qualitative methods within a study overall provides essential insight into how and why an intervention is effective,

For peer review only - http://bmjopen.bmj.com/site/about/guidelines.xhtml

if at all [18 19]. The purpose of this study was to gain an understanding of attitudes and beliefs among psychiatrists who had been part of the CODES Trial and were experienced in working with patients with DS, with particular emphasis on psychiatrists' views of other HCPs' ability and willingness to work with DS patients in the context of the NHS.

For peer review only

For peer review only - http://bmjopen.bmj.com/site/about/guidelines.xhtml

METHODS

Study population

Ten participants were purposively selected from the 29 psychiatrists involved in the CODES RCT to encompass the geographical distribution of the CODES Trial and the range of experience treating functional neurological disorders (FNDs), particularly DS. All participants were known to the wider CODES team prior to taking part in this qualitative study. CODES Trial grant holders were excluded to avoid study design-related bias. The psychiatrists were based at nine different NHS Trust tertiary or secondary mental health services across England with one based in a specialist neurological hospital. Recruitment took place between June and September 2017.

Data Collection

Those selected were initially contacted via email by HJ and invited to take part. Half of those approached had a prior working relationship with HJ within the CODES Trial. They were provided with an information sheet and a description of the aims of the project. If they were interested, a work place based face-to-face interview was scheduled at a time and date convenient for them. All participants provided informed consent to be interviewed. There were no refusals to participate. All interviews were conducted by HJ and recorded using an encrypted digital voice recorder to ensure data security and confidentiality. Interviews lasted between 41 – 96 minutes, covering the complete interview schedule.

Due to the nature of responses, it was not possible to determine the duration of responses solely covering the themes discussed in the current paper.

Interview Schedule

The interview schedule was developed by members of the CODES study team of which all authors were a part. The topics covered experiences of delivering the CODES SMC and involvement in the CODES RCT more generally (which will be reported elsewhere). In addition, topics covered the delivery of diagnosis, DS in the context of the NHS, and the challenges of the patient group, which are the focus of this paper. Participants were encouraged to give examples where possible and probing techniques were used to explore responses and elicit further detail where necessary [20] (see Supplementary file). The interview began with a series of questions about aspects of the trial processes which will be reported elsewhere. Although the topic guide focussed on involvement in the CODES trial and distinguished this way of working from more general issues of working with patients with DS, the

For peer review only - http://bmjopen.bmj.com/site/about/guidelines.xhtml

nature of responses meant that these topics often overlapped, and participants sometimes discussed issues relevant to multiple topics in a single answer. Participants readily elaborated with examples from their practise and experience outside of their involvement in the CODES RCT. During the stepwise coding and analysis of the interviews, several themes concerning the challenges of treating this patient group emerged. This inductive process inspired an analysis where the psychiatrists' accounts were used and contextualised through a specific focus on their views of other HCPs' attitudes to DS and their ability and willingness to work with patients with DS.

Data Analysis

The interviews were transcribed verbatim by members of the CODES research team. During the transcription process the interview data was anonymised. Completed transcripts were checked by HJ against the original recordings to ensure accuracy. The semi-structured interviews were analysed using thematic analysis. [21] This method was chosen rather than, for example, grounded theory, as our aim was to understand participants' professional views and methods of working with patients with DS rather than to develop theory. Three of the completed transcripts were chosen at random and coded initially by HJ, SF and another member of the research team. Emerging findings and preliminary themes were discussed in team meetings. HJ and SF then coded all 10 transcripts independently, using the qualitative data analysis software NVivo 11 (QSR International). NVivo allows the researcher to see how many interviews referred to a particular theme. Coding was done independently to allow for an organic and reflexive process. All content was grouped into categories to allow for the identification of patterns in the data. As each interview was analysed, new categories were added to the list and content was organized under each relevant category. Regular meetings were held to discuss agreements in coding and establish the parameters of each major theme. Major themes were established from the categories that contained the most substantial amount of data. Themes that had been identified by both coders were then combined, with subthemes being organized under the appropriate over-arching theme. We believe saturation had been reached since, as the interviews progressed, it was clear no new major themes were being elicited.

Patient and public involvement

The CODES Trial has a number of service users (i.e. individuals with DS or other relevant conditions) involved as members of its management committees, contributing to decisions about the running of the study and commenting on project outputs (e.g. papers).

RESULTS

For peer review only - http://bmjopen.bmj.com/site/about/guidelines.xhtml

Interviews from the 10 psychiatrists were analysed (see Table 1 for the psychiatrists' demographic characteristics). In general, there was a consistent level of agreement among participants on most topics covered. This made it straightforward to identify main themes and clearly convey the conclusions drawn from the clinicians. Though the topic guide elicited a broad range of themes, for the purposes of this paper we focused on those that had significant clinical implications. Other themes relating to the CODES Trial will be described elsewhere. Two main clinically relevant overarching themes emerged from the data: i) Other Healthcare Professionals and DS and (ii) Psychiatrists' Identified Challenges of Working with DS.

Table 1 *Psychiatrists' self-reported demographic information*

	N	%		N	%
**Age**			**Sub Specialist Accreditation**		
31-40	1	10%	Liaison Psychiatry	6	60%
41-50	8	80%	Neuropsychiatry	1	10%
51-60	1	10%	Both	3	30%
**Gender**			**Years of Experience**		
Female	5	50%	11-15	5	50%
Male	5	50%	16-20	2	20%
			21-25	1	10%
**Location**			26-30	2	20%
London	6	60%			
Rest of England	4	40%			

For peer review only

Other Healthcare Professionals (HCPs) and DS

HCPs ill-equipped to deal with DS

Psychiatrists reported that HCPs from other services often felt uncertain when dealing with DS patients or were not prepared to work with patients with functional neurological symptoms. Others felt that DS is a disorder that GPs should better understand. It was also reported that services would often contribute to the diagnostic confusion by continually mistaking DS for epilepsy, despite referrals stating otherwise;

> *"They would come back saying well, look, this is epilepsy, they need to be seeing a neurologist, or people would end up back on anticonvulsants"*
> *(Psychiatrist 09, Female, Liaison Psychiatry).*

For peer review only - http://bmjopen.bmj.com/site/about/guidelines.xhtml

The mention of seizures would often result in a panicked response from some primary care psychology services that meant patients could sense having something difficult to treat. Psychiatrists described patients often feeling other clinicians had not given a positive message about a DS diagnosis, with some GPs reportedly stating the need to be on an anticonvulsant simply at the mention of seizures. This continual reference to epilepsy by other professionals can have a negative impact on patients' progress,

> *"They have said, oh this sounds…you have epilepsy. I say don't say that, you're not qualified to say that, you know, you do your job, uh because that one word would put patients (pause) back, by a year or two or ten sessions."*
> *(Psychiatrist 06, Male, Neuropsychiatry)*

Psychiatrists would find that making DS referrals to psychology services would result in the referral being rejected unless patients had a comorbidity that psychologists felt they could treat;

> *"So, if you send a referral saying this person has dissociative seizures, will you see them, they will return the referral, so you have to say, "this person has dissociative seizures; however, they also have a very clear anxiety or panic disorder and that is what I would like you to work on" and then they will accept it."*
> *(Psychiatrist 03, Female, Liaison Psychiatry)*

This was reiterated throughout most of the interviews, with psychiatrists stating that local services would prefer to treat comorbidities rather than the DS themselves and where no comorbidity could be identified, services often rejected the referrals. The majority of the interviewees endorsed the view that psychiatrists were a key part of DS patient care. However, two of the 10 questioned whether it was necessary in all cases for a psychiatrist to be involved especially if the DS patient had no clear psychiatric co-morbidities. This approach seemed to be influenced both by their usual practice and service pressures at the two trusts.

The need for experience

One conclusion frequently drawn from psychiatrists' experiences with HCPs in other services was that, in order to diagnose and treat DS, the clinician needed to have a significant level of experience with the disorder and that treatment should be undertaken in a specialist setting,

For peer review only - http://bmjopen.bmj.com/site/about/guidelines.xhtml

"I sincerely believe that…it's not a condition which anybody or everybody can deal with and I don't think it should be dealt with at IAPT level"

(Psychiatrist 06, Male, Neuropsychiatry). (IAPT = Improving Access to Psychological Therapies services; the IAPT programme began in 2008 and aims to offer short-term evidence based psychological treatments for depression and anxiety in adults across England).

Delivering treatment in a specialist centre was described as not only helpful in terms of clinicians knowing how to work with DS, but would provide reassurance for patients that they were being seen by someone who is confident and experienced;

"I think it's one of those conditions where seeing people who know what it is, know what to do with it even if they can't promise to get it better it reduces everybody's anxiety levels about it" (Psychiatrist 09, Female, Liaison Psychiatry).

This sense of needing experience was also reported as helpful in enabling professionals to acknowledge the amount that can often be unknown about the causes and triggers for DS and for helping the patient embrace that as well.

Psychiatrists' Identified Challenges of Working with DS

Avoidance

Avoidance was viewed as a key area of difficulty for the DS patient group and was noted to take a number of forms across 9/10 interviews. Examples of avoidance were given in the interviews but these fall into two broad categories: "avoidance of help" and "avoidance of emotions". Avoidance of help included not reading information about DS even when handed this directly in an appointment and avoiding attending medical or therapy appointments. Avoidance of emotions included a desire to take medication rather than deal with difficult feelings and blocking emotions;

"Quite a lot of people may have blocked out so to speak, the more emotional side of things and try to get on with things…. if they start to go through a more open exploration of the issues this can be very emotionally distressing and suddenly their mood goes down…… a lot of patients will have to go through that turbulence in order to come out on the other end having felt the issues, recognised them and dealt with it"

(Psychiatrist 06, Male, Neuropsychiatry)

For peer review only - http://bmjopen.bmj.com/site/about/guidelines.xhtml

The seizures themselves were seen as potentially fitting into a pattern of avoidant behaviour as a defence against difficult emotions. Avoidant behaviour could present under the guise of other difficulties such as a reluctance to travel to appointments.

Linked to the theme of avoidance was emotional literacy. This was commented on by 9/10 interviewees as a key difficulty for the DS patient group. It follows that if a person lacks awareness of their own emotions they would struggle to express these to others. This lack of emotional awareness could then impede the ability to make links between life events and feelings whilst in treatment.

> *"No symptoms, happy go lucky kind of personality, I love my family, no trauma, no pain and those people are the hardest"*
> (Psychiatrist 10, Female, Liaison Psychiatry)

Some of the most challenging of the DS patient group were those for whom no trigger for the seizures could be identified. Sometimes even when it was very clear to the clinician that there was a current stressor (such as caring for a gravely ill partner) patients with DS might deny this was the case. This seemed to lead to feelings of frustration for the psychiatrists as they viewed patients with no identified psychological trigger harder to treat successfully.

Complex interpersonal relationships

Eight out of the 10 psychiatrists noted that a patient with DS may well struggle with relationships, both with people generally and within the clinician-patient relationship. This could be associated with difficulties in attachment with the DS patient becoming over-attached and then not wanting to engage with any other clinician or be discharged.

> *"She became quite attached; there were real attachment issues…. so, I only managed to discharge her as I said a few weeks ago…… she didn't connect with that person [CODES CBT therapist] and she created a split between that person and me and she was like I just want to come and see you, can't I just come and see you every two weeks?"*
> (Psychiatrist 08, Female Liaison Psychiatry)

Attempts at splitting were described as occurring not only between individual clinicians (as above) but also between services e.g., a specialist national service being "good" and all local services being "bad". This splitting and idealisation of one service or clinician could be accompanied by unrealistic

For peer review only - http://bmjopen.bmj.com/site/about/guidelines.xhtml

expectations that the psychiatrist would continue to see them indefinitely. A partner being in the room throughout every appointment can mean the patient is less able to be open about how they are feeling and certainly less likely to discuss any current relationship difficulties. A co-dependent relationship may also impact on motivation to recover as some people with DS have social circumstances in which getting better may feel riskier than remaining unwell. A couple of the psychiatrists went further and conceptualised some examples of factitious behaviours in patients (e.g. deliberately concealing medication non-adherence from family and clinicians) as driven by a profound need to be looked after.

DISCUSSION

We present here the experiences and views of the psychiatrists involved in an RCT for patients with DS. The characteristics of the nested group were similar to the clinicians involved in the trial as a whole in relation to age, gender, ethnicity and experience working with DS. Participants generally expressed concordant views across the range of interview questions, suggesting that issues surrounding DS are very apparent to the professionals working closely with this population. Views broadly support previous research that describes DS patients as a heterogeneous population with complex presentations and demanding HCP input, [8 10 21] as well as the need for an improvement in education and awareness of DS by HCPs. [22]

Participants believed that interventions by other HCPs at times made their own work with this patient group more difficult. They identified a knowledge gap surrounding DS among other HCPs. Previous research has found that HCPs from a variety of backgrounds often have very different perceptions regarding DS, [23] which seems to be a pervasive problem at all levels of health services in the UK, from GPs and primary care services to Community Mental Health Trusts and Emergency Departments. While we cannot report here on the perceptions of other HCPs involved in the CODES Trial, what is perhaps most significant is the current participants' observation that this knowledge gap can at times have a detrimental effect on patients' prognosis, with one participant noting that the mention of epilepsy and AED treatment can set a patient's progress back significantly. Rawlings and Reuber's recent review[10] raised concerns not only about HCPs' DS knowledge gap but also negative attitudes towards the condition. It is of concern that negative attitudes towards the DS patient group may be created or reinforced by HCPs currently in practice when training junior staff. [10] Dworetzky [24] reported that epilepsy specialists in the USA when teaching junior staff about DS tend to focus on the cost of care and misuse of services caused by DS. Negative clinician attitudes towards DS patients themselves

For peer review only - http://bmjopen.bmj.com/site/about/guidelines.xhtml

For peer review only

did not emerge as a theme here, perhaps because our group of psychiatrists have chosen to work with this patient group.

Referring to epilepsy can be damaging to patients with DS as it contributes to diagnostic confusion in a number of ways. If they have been diagnosed with DS already, it may cause them to doubt whether the diagnosis is correct. It may initiate or strengthen a belief that they in fact have epilepsy, despite diagnostic evidence (e.g.; video-electroencephalography (vEEG) telemetry results) and clinical opinion to the contrary. [25] It also means that often DS patients are treated with potentially harmful anti-epileptic drugs (AEDs) with serious side effects despite them having no medical benefit and this can lead to serious iatrogenic harm. [26] It would be helpful for more specific epilepsy-related guidance to be developed for HCPs with regards to reducing AEDs and handling a misdiagnosis of epilepsy so as to avoid any setbacks in recovery. HCPs should be made aware of the clinical significance that simply mentioning epilepsy and AEDs can have on a patient and this should be highlighted in any future educational resources.

The International League Against Epilepsy (ILAE) PNES Task Force produced a special report describing the minimum requirements for a diagnosis of DS[27]. It would be beneficial for those working with DS to become familiar with these guidelines so that they can clearly convey their diagnostic reasoning to the patient and can discuss the features of DS and the characteristics distinguishing DS from epilepsy more confidently. In addition to this detailed account of the diagnosis, LaFrance et al. [25] also produced a comprehensive overview of the management of DS patients, encompassing diagnosis, treatment and maintaining engagement. Whilst it is argued that the management of DS requires expertise, the guidelines are accessible and can be used as a helpful tool for non-experts to familiarize themselves with the important elements for interacting with this patient group.

There were some discordant views surrounding which services and professionals should be able to work with DS. Some participants described the need for more specialist services to be made available and for psychiatric training to cover functional neurological symptoms in greater depth. However, some participants stressed the necessity for GPs and primary care clinicians to also become more familiar with DS. Despite this contrast, the consensus remains that more education and awareness are required for all HCPs regardless of their speciality. This supports previous research that has reported the need for more clinicians to be comfortable with treating all FNDs, particularly DS. [28] Encouragingly going forward even a brief training intervention for medical students and doctors' improved confidence levels and diagnostic accuracy when working with DS. [29 30]

For peer review only - http://bmjopen.bmj.com/site/about/guidelines.xhtml

To address this issue, better resources and educational materials need to be made available for those who are likely to be working with DS, both for future clinicians and clinicians currently in practice. Rommelfanger et al. [22] described a level of "professional isolation" often felt among care providers working with functional symptoms due to a distinct lack of formalised training and reported on the need for a shift in priorities to support clinicians working with patients with FNDs. This could start as early as medical school but should also involve the development of sufficient resources to support the multidisciplinary approach that is often required to treat FNDs.

Avoidant behaviours could be divided into two categories of "avoidance of help" and "avoidance of emotions". Linked to avoidance was the subtheme of emotional literacy, difficulty feeling and expressing emotions. These findings support previous quantitative research on alexithymia in patients with DS, where Bewley et al. [31] found DS patients had significantly greater difficulty identifying feelings than healthy controls. More recently, Uliaszek et al. [32] found significant emotion dysregulation among DS patients when compared with a control group. This may have important implications for future therapeutic developments, by incorporating elements of effective treatments for alexithymia from other treatment models (e.g. mindfulness-based therapy for alexithymia). [33] Previous research also found evidence of experiential avoidance [34] and avoidant coping styles [35] among the DS patient population. Given these findings, it is important to establish what impact this avoidant behaviour may have on treatment outcomes and what can be done to mitigate any negative impacts. Some participants in the present study reported positive progress in openly identifying the patients' avoidant behaviour and providing the opportunity to address it constructively. Treatment approaches for DS should make provision for tackling avoidance directly. [16 36] In addition, for a subset of the patient group, no trigger for DS is identified and these patients tended to be particularly difficult to treat from the point of explaining the diagnosis onwards. Working with this complex patient group effectively clearly needs experience and knowledge of the condition.

Our study has a number of limitations. The study has a small sample size of 10 psychiatrists currently working at healthcare centres around England. Psychiatrists working in Scotland and Wales are not represented in this group. As participants were all involved in the CODES Trial and knew the interviewer or other members of the research team, it is possible this influenced their responses. However, as the interview did not solely focus on the CODES Trial, other aspects may be less likely to have been affected by prior working relationships.

For peer review only - http://bmjopen.bmj.com/site/about/guidelines.xhtml

It is possible that the DS patient sample in the CODES Trial, who will have partly influenced the current psychiatrists' views, is not fully representative of the DS patient population in general. It is also likely that there is a self-selection bias for those presenting to psychiatrists as many DS patients may reject a psychiatric diagnosis and thus do not attend psychiatric appointments. [37] The disparity in services may mean that some patients simply do not have access to a specialist psychiatrist and are referred back to their GP. [38] We will report elsewhere an exploration of the views of patients with DS involved in the CODES Trial to triangulate findings and maximise our understanding of working and living with DS. All participants in this current study were specialist psychiatrists and therefore not representative of the population of psychiatrists more generally in the NHS across the UK. However, due to the organisation of care in the NHS, DS patients are not usually seen by general psychiatrists. Therefore, our sample is representative of those clinicians who are most likely to provide direct clinical contact with the DS patient population. It is possible that talking about the challenges of the patient group would have led the respondents to think more about their perception of the difficulties of working with this patient group and may have influenced the nature of the emerging themes.

In terms of strengths, to our knowledge, our study was the first qualitative study focusing solely on psychiatrists' perspectives of working with patients with DS. Prior published qualitative studies [39 40] interviewed healthcare professionals from a variety of backgrounds. However, McMillan et al.'s [39] large sample (74 interviews) only included epilepsy staff such as neurologists, EEG technicians and epilepsy nurses and no mental health care providers. In du Toit & Pretorius's [40] study, only three of 15 people interviewed were psychiatrists. Given that DS is classified as a mental health disorder (DSM5, [41]) and a dissociative disorder (ICD-10 [42]) and will predominantly be handled by mental health clinicians, it seems important to explore the views of those working with the DS population in the appropriate clinical context. In addition, it is likely that the current sample is more representative of the clinicians most likely to be working with DS in developed countries, and not limited to a military clinical environment. [39]

CONCLUSION

Qualitative findings suggest that patients with DS are a complex and at times challenging population that requires intervention from experienced clinicians familiar with the condition. Significantly, intervention can be made more difficult if not provided in an informed and experienced manner. Our findings have important implications for medical and allied professional training with regards to FNDs

For peer review only - http://bmjopen.bmj.com/site/about/guidelines.xhtml

in order for clinicians to be better equipped to recognise and handle the challenges that come with treating DS. It is also hoped that a greater evidence base for treatments for patients with DS will help eradicate the variability within healthcare provision.

Acknowledgements: The authors would like to thank the psychiatrists who were willing to be interviewed for this study given their busy schedules. The authors also thank Julie Read for her assistance with the project.

Author contributions: HJ, IP, JM and LHG contributed to the development of the topic guide. HJ carried out all data collection. HJ and SF transcribed the data, carried out the analysis and led the writing of the paper. JM and IP reviewed drafts of the paper. LHG oversaw the project, contributed to the writing of the paper and reviewed successive drafts. All authors approved the final version of the submitted text.

Funding statement: This paper describes independent research funded by the National Institute for Health Research (Health Technology Assessment programme, 12/26/01, COgnitive behavioural therapy vs standardised medical care for adults with Dissociative non-Epileptic Seizures: A multicentre randomised controlled trial (CODES)). LHG receives salary support from the National Institute for Health Research (NIHR) Maudsley Biomedical Research Centre at the South London and Maudsley NHS Foundation Trust and King's College London. The views expressed in this publication are those of the authors and not necessarily those of the NHS, the NIHR or the Department of Health.

Competing interests: LHG and JM report the grant from the NIHR HTA for the conduct of the study. None of the other authors have competing interests to declare.

Data sharing statement: Contact the corresponding author with queries.

Ethics approval: The study was approved by the NHS Health Research Authority, London – Camberwell St Giles Research Ethics Committee (reference number 13/LO/1595). Written informed consent was obtained from all study participants. Study registration: Current Controlled Trials: ISRCTN05681227; ClinicalTrials.gov: NCT02325544.

For peer review only - http://bmjopen.bmj.com/site/about/guidelines.xhtml

REFERENCES

1. Duncan R, Razvi S, Mulhern S. Newly presenting psychogenic nonepileptic seizures: Incidence, population characteristics, and early outcome from a prospective audit of a first seizure clinic. *Epilepsy Behav* 2011;20(2):308-11. doi: 10.1016/j.yebeh.2010.10.022
2. Kanemoto K, LaFrance WC, Duncan R, et al. PNES around the world: Where we are now and how we can close the diagnosis and treatment gaps—an ILAE PNES Task Force report. *Epilepsia Open* 2017;2(3):307-16. doi: doi:10.1002/epi4.12060
3. Asadi-Pooya AA, Sperling MR. Epidemiology of psychogenic nonepileptic seizures. *Epilepsy Behav* 2015;46:60-65. doi: https://doi.org/10.1016/j.yebeh.2015.03.015
4. Kerr MP, Mensah S, Besag F, et al. International consensus clinical practice statements for the treatment of neuropsychiatric conditions associated with epilepsy. *Epilepsia* 2011;52(11):2133-38. doi: 10.1111/j.1528-1167.2011.03276.x
5. Angus-Leppan H. Diagnosing epilepsy in neurology clinics: A prospective study. *Seizure* 2008;17(5):431-36. doi: 10.1016/j.seizure.2007.12.010
6. Lesser RP. Psychogenic seizures. *Neurology* 1996;46(6):1499-507.
7. Worsely C, Whitehead K, Kandler R, et al. Illness perceptions of health care workers in relation to epileptic and psychogenic nonepileptic seizures. *Epilepsy Behav* 2011;20(4):668-73. doi: https://doi.org/10.1016/j.yebeh.2011.01.029
8. Hingray C, El-Hage W, Duncan R, et al. Access to diagnostic and therapeutic facilities for psychogenic nonepileptic seizures: An international survey by the ILAE PNES Task Force. *Epilepsia* 2018;59(1):203-14. doi: doi:10.1111/epi.13952
9. Kanaan RA, Armstrong D, Wessely SC. Neurologists' understanding and management of conversion disorder. *J Neurol Neurosurg Psychiatry* 2011;82(9):961-6. doi: http://dx.doi.org/10.1136/jnnp.2010.233114
10. Rawlings GH, Reuber M. Health care practitioners' perceptions of psychogenic nonepileptic seizures: A systematic review of qualitative and quantitative studies. *Epilepsia* 2018;59(6):1109-23. doi: doi:10.1111/epi.14189
11. Karterud HN, Knizek BL, Nakken KO. Changing the diagnosis from epilepsy to PNES: Patients' experiences and understanding of their new diagnosis. *Seizure* 2010;19(1):40-46. doi: http://dx.doi.org/10.1016/j.seizure.2009.11.001
12. Rawlings GH, Reuber M. What patients say about living with psychogenic nonepileptic seizures: A systematic synthesis of qualitative studies. *Seizure* 2016;41:100-11. doi: http://dx.doi.org/10.1016/j.seizure.2016.07.014
13. Shneker BF, Elliott JO. Primary care and emergency physician attitudes and beliefs related to patients with psychogenic nonepileptic spells. *Epilepsy Behav* 2008;13(1):243-47. doi: https://doi.org/10.1016/j.yebeh.2008.03.001
14. O'Sullivan SS, Sweeney BJ, McNamara B. The opinion of the general practitioner toward clinical management of patients with psychogenic nonepileptic seizures. *Epilepsy Behav* 2006;8(1):256-60. doi: https://doi.org/10.1016/j.yebeh.2005.09.013
15. Nielsen ES, Wichaidit BT, Østergaard JR, et al. Paediatricians' attitudes to and management of functional seizures in children. *European Journal of Paediatric Neurology* 2018;22(5):774-81. doi: 10.1016/j.ejpn.2018.05.007
16. Goldstein LH, Mellers JDC, Landau S, et al. COgnitive behavioural therapy vs standardised medical care for adults with Dissociative non-Epileptic Seizures (CODES): A multicentre randomised controlled trial protocol. *BMC Neurology* 2015;15(1) doi: http://dx.doi.org/10.1186/s12883-015-0350-0
17. Lewin S, Glenton C, Oxman AD. Use of qualitative methods alongside randomised controlled trials of complex healthcare interventions: methodological study. *BMJ* 2009;339 doi: 10.1136/bmj.b3496

For peer review only - http://bmjopen.bmj.com/site/about/guidelines.xhtml

18. Möhler R, Köpke S, Meyer G. Criteria for Reporting the Development and Evaluation of Complex Interventions in healthcare: revised guideline (CReDECI 2). *Trials* 2015;16(1):204. doi: 10.1186/s13063-015-0709-y
19. Campbell M, Fitzpatrick R, Haines A, et al. Framework for design and evaluation of complex interventions to improve health. *BMJ* 2000;321 doi: 10.1136/bmj.321.7262.694
20. Rubin H, Rubin I. Qualitative interviewing: A method to the madness: Thousand Oaks, CA: Sage Publications, 1995.
21. Braun V, Clarke V. Using thematic analysis in psychology. *Qualitative Research in Psychology* 2006;3(2):77-101. doi: 10.1191/1478088706qp063oa
22. Rommelfanger KS, Factor SA, LaRoche S, et al. Disentangling Stigma from Functional Neurological Disorders: Conference Report and Roadmap for the Future. *Front Neurol* 2017;8(106) doi: 10.3389/fneur.2017.00106
23. Whitehead K, Reuber M. Illness perceptions of neurologists and psychiatrists in relation to epilepsy and nonepileptic attack disorder. *Seizure* 2012;21(2):104-9.
24. Dworetzky BA. What Are We Communicating When We Present the Diagnosis of PNES? *Epilepsy Curr* 2015;15(6):353-57. doi: 10.5698/1535-7511-15.6.353
25. Lafrance Jr WC, Reuber M, Goldstein LH. Management of psychogenic nonepileptic seizures. *Epilepsia* 2013;54:53-67. doi: http://dx.doi.org/10.1111/epi.12106
26. Reuber M, Elger CE. Psychogenic nonepileptic seizures: Review and update. *Epilepsy and Behavior* 2003;4(3):205-16.
27. Lafrance Jr WC, Baker GA, Duncan R, et al. Minimum requirements for the diagnosis of psychogenic nonepileptic seizures: A staged approach: A report from the International League Against Epilepsy Nonepileptic Seizures Task Force. *Epilepsia* 2013;54(11):2005-18. doi: http://dx.doi.org/10.1111/epi.12356
28. Gardiner P, MacGregor L, Carson A, et al. Occupational therapy for functional neurological disorders: a scoping review and agenda for research. *CNS Spectrums* 2017;23(3):205-12. doi: 10.1017/S1092852917000797 [published Online First: 11/27]
29. O'Sullivan SS, Redwood RI, Hunt D, et al. Recognition of psychogenic non-epileptic seizures: a curable neurophobia? *Journal of Neurology, Neurosurgery & Psychiatry* 2013;84(2):228-31. doi: 10.1136/jnnp-2012-303062
30. Seneviratne U, Ding C, Bower S, et al. Video-based training improves the accuracy of seizure diagnosis. *Journal of Neurology, Neurosurgery & Psychiatry* 2014 doi: 10.1136/jnnp-2013-306618
31. Bewley J, Murphy PN, Mallows J, et al. Does alexithymia differentiate between patients with nonepileptic seizures, patients with epilepsy, and nonpatient controls? *Epilepsy and Behavior* 2005;7(3):430-37.
32. Uliaszek AA, Prensky E, Baslet G. Emotion regulation profiles in psychogenic non-epileptic seizures. *Epilepsy Behav* 2012;23(3):364-69. doi: https://doi.org/10.1016/j.yebeh.2012.01.009
33. Norman H, Marzano L, Coulson M, et al. Effects of mindfulness-based interventions on alexithymia: a systematic review. *Evidence Based Mental Health* 2018
34. Dimaro LV, Dawson DL, Roberts NA, et al. Anxiety and avoidance in psychogenic nonepileptic seizures: the role of implicit and explicit anxiety. *Epilepsy & Behavior* 2014;33:77-86. doi: http://dx.doi.org/10.1016/j.yebeh.2014.02.016
35. Goldstein LH, Drew C, Mellers J, et al. Dissociation, hypnotizability, coping styles and health locus of control: Characteristics of pseudoseizure patients. *Seizure* 2000;9(5):314-22.
36. Goldstein LH, Chalder T, Chigwedere C, et al. Cognitive-behavioral therapy for psychogenic nonepileptic seizures A pilot RCT. *Neurology* 2010;74(24):1986-94.
37. Carton S, Thompson PJ, Duncan JS. Non-epileptic seizures: patients' understanding and reaction to the diagnosis and impact on outcome. *Seizure* 2003;12(5):287-94.

For peer review only - http://bmjopen.bmj.com/site/about/guidelines.xhtml

38. Agrawal N, Fleminger S, Ring H, et al. Neuropsychiatry in the UK: National survey of existing service provision. *Psychiatric Bulletin* 2008;32(8):288-91. doi: http://dx.doi.org/10.1192/pb.bp.107.018424
39. McMillan KK, Pugh MJ, Hamid H, et al. Providers' perspectives on treating psychogenic nonepileptic seizures: Frustration and hope. *Epilepsy and Behavior* 2014;37:276-81. doi: http://dx.doi.org/10.1016/j.yebeh.2014.07.001
40. du Toit A, Pretorius C. Psychogenic nonepileptic seizures: Namibian healthcare providers' perceptions and frustrations. *Seizure* 2017;50:43-52. doi: https://doi.org/10.1016/j.seizure.2017.06.004
41. American Psychiatric Association. Diagnostic and statistical manual of mental disorders Fifth edition. United States: American Psychiatric Association; 2013.
42. World Health Organization. The ICD-10 Classification of Mental and Behavioural Disorders: Clinical Descriptions and Diagnostic Guidelines: World Health Organization 1992.

For peer review only

For peer review only - http://bmjopen.bmj.com/site/about/guidelines.xhtml

Supplementary file

Psychiatrists' interview schedule from which current themes were elicited (material related to the conduct of the CODES Trial to be reported elsewhere)

**Psychiatrists Interview Schedule**
**Section 1: Background and Specific Issues relating to the CODES RCT**<br><br>**How does CODES standardised medical care (SMC) differ from the techniques you would usually use to treat patients with DS?**<br><br>**If SMC is shown to reduce seizures, what do you think will be most difficult about making SMC standard across services?**<br><br>**Were there any parts of the CODES SMC approach that proved more challenging?**<br><br>**How did you manage significant deterioration in a participant's mental health during their time in the CODES study?**<br><br>**How did you feel about NOT referring to other types of therapy whilst a participant was in CODES?**<br><br>**Section 2: Experience of the Intervention**<br><br>**Did the way patients engaged with standardised medical care (SMC) seem to change over time?**<br><br>**Were there any 'lightbulb moments' in the course of SMC where patients appeared to have a sudden understanding of their condition?**<br><br>**Section 3: Individual Psychological, Social or Health-Related Differences and Impact on Treatment**<br><br>**Do you think that there were any factors that may have affected patients understanding of their diagnosis?**<br><br>**Were there any patients who may have been more suitable than others to receive SMC alone? If so, what distinguished these types of clients?**<br><br>**Were there issues that you had to address in order to improve engagement? Or were there any barriers to patients engaging with SMC?**

For peer review only

Could sessions ever become side-tracked/derailed by other issues? E.g.: social issues, safeguarding or health-related concerns?

For peer review only

Consolidated criteria for reporting qualitative studies (COREQ): 32-item checklist

Developed from:
Tong A, Sainsbury P, Craig J. Consolidated criteria for reporting qualitative research (COREQ): a 32-item checklist for interviews and focus groups. *International Journal for Quality in Health Care*. 2007. Volume 19, Number 6: pp. 349 – 357

YOU MUST PROVIDE A RESPONSE FOR ALL ITEMS. ENTER N/A IF NOT APPLICABLE

No. Item	Guide questions/description	Reported on Page #
**Domain 1: Research team and reflexivity**		
Personal Characteristics		
1. Inter viewer/facilitator	Which author/s conducted the **interviews** or focus group?	Methods, Page 5
2. Credentials	What were the researcher's credentials? E.g. PhD, MD	Authors, Page 1
3. Occupation	What was their occupation at the time of the study?	Methods, Page 5, where we state that The interview schedule was developed by members of the CODES study team, of which all authors were members.
4. Gender	Was the researcher male or female?	Authors list. Page 1
5. Experience and training	What experience or training did the researcher have?	Author JM is a qualitative research expert and supervised the project.
Relationship with participants		
6. Relationship established	Was a relationship established prior to study commencement?	Member of the CODES study research team had prior involvement with participants, as all interviewees are involved in this study. Participants, Page 5.
7. Participant knowledge of the interviewer	What did the participants know about the researcher? e.g. personal goals, reasons for doing the research	See above.
8. Interviewer characteristics	What characteristics were reported about the inter viewer/facilitator? e.g. Bias, assumptions, reasons and interests in the	N/A We have not reported this as we

For peer review only

	research topic	are not undertaking IPA.
**Domain 2: study design**		
Theoretical framework		
9. Methodological orientation and Theory	What methodological orientation was stated to underpin the study? e.g. grounded theory, discourse analysis, ethnography, phenomenology, content analysis	Thematic Analysis. Methods, Page 5.
Participant selection		
10. Sampling	How were participants selected? e.g. **purposive**, convenience, consecutive, snowball	Methods, Page 5.
11. Method of approach	How were participants approached? e.g. face-to-face, telephone, mail, **email**	Methods, Page 5
12. Sample size	How many participants were in the study?	Methods, Page 5
13. Non-participation	How many people refused to participate or dropped out? Reasons?	No refusals. N/A.
Setting		
14. Setting of data collection	Where was the data collected? e.g. home, clinic, workplace	Face to face. Methods, page 5
15. Presence of non-participants	Was anyone else present besides the participants and researchers?	N/A
16. Description of sample	What are the important characteristics of the sample? e.g. demographic data, date	Results, Demographic Table Page 6
Data collection		
17. Interview guide	Were questions, prompts, guides provided by the authors? Was it pilot tested?	Methods, page 5 Prompts & Probes used. Not pilot tested.
18. Repeat interviews	Were repeat inter views carried out? If yes, how many?	No repeat interviews conducted. N/A.
19. Audio/visual recording	Did the research use audio or visual recording to collect the data?	Methods, Page 5
20. Field notes	Were field notes made during and/or after the inter view or focus group?	N/A
21. Duration	What was the duration of the inter views or focus group?	Methods, Page 5
22. Data saturation	Was data saturation discussed?	Yes at the end of the Data Analysis section
23. Transcripts returned	Were transcripts returned to participants for comment and/or correction?	N/A
**Domain 3: analysis and findings**		
Data analysis		
24. Number of data coders	How many data coders coded the data?	Methods, Page 5
25. Description of the coding tree	Did authors provide a description of the coding tree?	Methods, Page 5 & Page 6 describes the coding process

For peer review only

		but no coding tree is given.
26. Derivation of themes	Were themes identified in advance or derived from the data?	Derived from the data. Methods, Page 6
27. Software	What software, if applicable, was used to manage the data?	NVivo 11. Methods, Page 5
28. Participant checking	Did participants provide feedback on the findings?	N/A
Reporting		
29. Quotations presented	Were participant quotations presented to illustrate the themes/findings? Was each quotation identified? e.g. participant number	Results, Pages 6-10 All participants allocated a number.
30. Data and findings consistent	Was there consistency between the data presented and the findings?	Results, Pages 6-10 Discussion, pages 10-14.
31. Clarity of major themes	Were major themes clearly presented in the findings?	Results, Pages 6-10
32. Clarity of minor themes	Is there a description of diverse cases or discussion of minor themes?	Results, Pages 6-10 Sub-themes discussed. High level of agreement between participants. Strongly endorsed themes.

Once you have completed this checklist, please save a copy and upload it as part of your submission. When requested to do so as part of the upload process, please select the file type: *Checklist*. You will NOT be able to proceed with submission unless the checklist has been uploaded. Please DO NOT include this checklist as part of the main manuscript document. It must be uploaded as a separate file.

For peer review only
